# Hypervirulent Klebsiella pneumoniae Causing Neonatal Bloodstream Infections: Emergence of NDM-1-Producing Hypervirulent ST11-K2 and ST15-K54 Strains Possessing pLVPK-Associated Markers

**DOI:** 10.1128/spectrum.04121-22

**Published:** 2023-02-08

**Authors:** Subhankar Mukherjee, Punyasloke Bhadury, Shravani Mitra, Sharmi Naha, Bijan Saha, Shanta Dutta, Sulagna Basu

**Affiliations:** a Division of Bacteriology, ICMR-National Institute of Cholera and Enteric Diseases, Kolkata, West Bengal, India; b Integrative Taxonomy and Microbial Ecology Research Group, Department of Biological Sciences, Indian Institute of Science Education and Research Kolkata, Mohanpur, Nadia, West Bengal, India; c Department of Neonatology, Institute of Post-Graduate Medical Education & Research and SSKM Hospital, Kolkata, West Bengal, India; Ohio State University

**Keywords:** *Klebsiella pneumoniae*, neonatal sepsis, antibiotic resistance, carbapenem resistance, hypervirulence, India

## Abstract

Klebsiella pneumoniae is a major cause of neonatal sepsis. Hypervirulent Klebsiella pneumoniae (hvKP) that cause invasive infections and/or carbapenem-resistant hvKP (CR-hvKP) limit therapeutic options. Such strains causing neonatal sepsis have rarely been studied. Characterization of neonatal septicemic hvKP/CR-hvKP strains in terms of resistance and virulence was carried out. Antibiotic susceptibility, molecular characterization, evaluation of clonality, *in vitro* virulence, and transmissibility of carbapenemase genes were evaluated. Whole-genome sequencing (WGS) and mouse lethality assays were performed on strains harboring pLVPK-associated markers. About one-fourth (26%, 28/107) of the studied strains, leading to mortality in 39% (11/28) of the infected neonates, were categorized as hvKP. hvKP-K2 was the prevalent pathotype (64.2%, 18/28), but K54 and K57 were also identified. Most strains were clonally diverse belonging to 12 sequence types, of which ST14 was most common. Majority of hvKPs possessed virulence determinants, strong biofilm-forming, and high serum resistance ability. Nine hvKPs were carbapenem-resistant, harboring *bla*_NDM-1_/*bla*_NDM-5_ on conjugative plasmids of different replicon types. Two NDM-1-producing high-risk clones, ST11 and ST15, had pLVPK-associated markers (*rmpA*, *rmpA2*, *iroBCDEN*, *iucABCDiutA*, and *peg-344*), of which one co-transferred the markers along with *bla*_NDM-1_. The 2 strains revealed high inter-genomic resemblance with the other hvKP reference genomes, and were lethal in mouse model. To the best of our knowledge, this study is the first to report on the NDM-1-producing hvKP ST11-K2 and ST15-K54 strains causing fatal neonatal sepsis. The presence of pLVPK-associated markers and *bla*_NDM-1_ in high-risk clones, and the co-transmission of these genes via conjugation calls for surveillance of these strains.

**IMPORTANCE** Klebsiella pneumoniae is a leading cause of sepsis in newborns and adults. Among the 2 major pathotypes of K. pneumoniae, classical (cKP) and hypervirulent (hvKP), hvKP causes community-acquired severe fatal invasive infections in even healthy individuals, as it possesses several virulence factors. The lack of comprehensive studies on neonatal septicemic hvKPs prompted this work. Nearly 26% diverse hvKP strains were recovered possessing several resistance and virulence determinants. The majority of them exhibited strong biofilm-forming and high serum resistance ability. Nine of these strains were also carbapenem (last-resort antibiotic)-resistant, of which 2 high-risk clones (ST11-K2 and ST15-K54) harbored markers (pLVPK) noted for their virulence, and were lethal in the mouse model. Genome-level characterization of the high-risk clones showed resemblance with the other hvKP reference genomes. The presence of transmissible carbapenem-resistant gene, *bla*_NDM_, along with pLVPK-markers calls for vigilance, as most clinical microbiology laboratories do not test for them.

## INTRODUCTION

Klebsiella pneumoniae is an opportunistic nosocomial pathogen, capable of causing several infecting syndromes in both adults and neonates. It is also the primary cause of neonatal sepsis in lower-middle-income countries (1, 2). Moreover, the global dissemination of carbapenem-resistant K. pneumoniae (CRKP) has created a substantial public-health concern (3). Among the carbapenemases, the New Delhi metallo-β-lactamase gene (*bla*_NDM_) is the most notorious due to the wide-spectrum of antimicrobial-hydrolyzing ability, presence in diverse conjugative plasmids and sequence types (STs), and the ability to cause large hospital-mediated outbreaks (4–7). Of the 43 NDM-variants that have been identified so far (https://www.ncbi.nlm.nih.gov/pathogens/refgene/#blaNDM), NDM-1 is the most widely disseminated, and exhibits significant resistance to clinically relevant β-lactams (4). Thus, these strains have been designated as an urgent threat to human health (8, 9).

To date, two major pathotypes of K. pneumoniae have been detected in clinical contexts: classical K. pneumoniae (cKP) and hypervirulent K. pneumoniae (hvKP). In contrast to the highly antimicrobial-resistant (AMR) cKP populations that primarily cause hospital-acquired infections, hvKP, the recently emerging pathotype can cause various community-acquired invasive fatal infections in immunocompetent individuals (10). Unlike cKP, hvKP can effectively sequester iron, often harbor plasmid-borne virulence factors (*rmpA*, *rmpA2*, *iro*, and *iuc*), and are frequently associated with several capsular types (K1, K2, K5, K20, K54, and K57) (11, 12). Though most hvKP strains have thick capsular polysaccharide and are hypermucoviscous, it should be noted that hypervirulence attributes of K. pneumoniae may not always be associated with hypermucoviscosity (13).

Since the emergence of hvKP almost 4 decades ago, most reported strains belonged to antibiotic-sensitive populations. However, a changing scenario of antibiotic-resistant hvKP has now been observed in clinical K. pneumoniae after the initial description of hvKP. Carbapenem-resistant hvKP (CR-hvKP) pathotypes are now frequently reported, and the clinical landscape of hvKP is altering dramatically (12, 14).

The occurrence of CR-hvKP in clinical settings has driven several recent studies (15, 16), as CR-hvKP pathotypes have the potential to be the ‘next generation super-bug’ (10). Although characterization of hvKP strains causing infections in adult population has been investigated, a detailed characterization of neonatal septicemic hvKP/CR-hvKP strains in terms of resistance and virulence is still lacking. The vulnerability of this population with limitations in therapeutic options, poses a greater challenge for clinicians. With increasing numbers of carbapenem-resistant neonatal infections, particularly where the causative organism harbors the *bla*_NDM_, an understanding of the burden of disease due to CR-hvKP is crucial in the neonatal population. Thus, hvKP/CR-hvKP strains recovered from septicemic neonates were investigated in terms of resistance, virulence, and transmission of the *bla*_NDM_ gene. This study also included genome-level characterization of selected CR-hvKP strains.

## RESULTS

### Antimicrobial susceptibility testing.

Among the recovered 107 K. pneumoniae, >50% of strains were resistant to 9 different antimicrobials (Fig. S1). Twenty-nine hvKP strains were detected, where 28 of them are characterized here, as previous work on CR-hvKP strain EN5275 (ST23-K1) has already been published (17).

Most of the recovered hvKPs were resistant to cephalosporin, cephamycin, monobactam, aminoglycosides, fluoroquinolone, and trimethoprim-sulfamethoxazole (Table 1). Among the studied hvKPs, only 32.1% (9/28) strains were detected as metallo-β-lactamase producers, resistant to carbapenems (MICs ranged from 8 to 64 mg/L), and were categorized as CR-hvKP.

**TABLE 1:** Microbiological features of recovered neonatal septicemic carbapenem-susceptible and carbapenem-resistant hvKP strains

Variable	*n* (%)^*b*^			*P* value
	Total, *n* = 28 (100%)	CS-hvKP, *n* = 19 (67.8%)	CR-hvKP, *n* = 9 (32.1%)	
AMR phenotypes (no. of strains resistant to particular antibiotics)				
Piperacillin	26 (92.8)	17 (89.4)	9 (100)	1.000
Cefotaxime	23 (82.1)	14 (73.6)	9 (100)	0.769
Cefoxitin	13 (46.4)	4 (21)	9 (100)	0.042^*a*^
Aztreonam	21 (75)	12 (63.1)	9 (100)	0.552
Meropenem	9 (32.1)	0 (0.0)	9 (100)	<0.001^*a*^
Amikacin	15 (53.5)	6 (31.5)	9 (100)	0.108
Gentamicin	14 (50)	7 (36.8)	7 (77.7)	0.322
Ciprofloxacin	23 (82.1)	14 (73.6)	9 (100)	0.769
Trimethoprim/Sulfamethoxazole	23 (82.1)	14 (73.6)	9 (100)	0.769
AMR genotypes				
β-lactams				
*bla*_TEM_	16 (57.1)	9 (47.3)	7 (77.7)	0.552
*bla*_SHV_	21 (75)	13 (68.4)	8 (88.8)	0.765
*bla*_OXA_	14 (50)	7 (36.8)	7 (77.7)	0.322
*bla*_CTX-M-15_	20 (71.4)	11 (57.8)	9 (100)	0.384
*bla*_AmpC_	8 (28.5)	3 (15.7)	5 (55.5)	0.216
*bla*_NDM-1_	7 (25)	0 (0.0)	7 (77.7)	0.001^*a*^
*bla*_NDM-5_	2 (7.1)	0 (0.0)	2 (22.2)	0.126
*bla*_OXA-232_	1 (3.5)	0 (0.0)	1 (11.1)	0.344
Aminoglycosides				
*armA*	2 (7.1)	0 (0.0)	2 (22.2)	0.126
*rmtB*	1 (3.5)	0 (0.0)	1 (11.1)	0.344
*rmtC*	3 (10.7)	0 (0.0)	3 (33.3)	0.048^*a*^
*aac(6′)-ib*	12 (42.8)	3 (15.7)	9 (100)	0.017^*a*^
Quinolone resistance genes				
*qnrB1*	16 (57.1)	10 (52.6)	6 (66.6)	0.750
*qnrS1*	9 (32.1)	2 (10.5)	7 (77.7)	0.023^*a*^
*oqxA*	21 (75)	12 (63.1)	9 (100)	0.552
*oqxB*	21 (75)	12 (63.1)	9 (100)	0.552
*aac(6′)-ib-cr*	17 (60.7)	10 (52.6)	7 (77.7)	0.748
Capsular types				
K2	18 (64.2)	12 (63.1)	6 (66.6)	1.000
K20	1 (3.5)	0 (0.0)	1 (11.1)	0.344
K54	5 (17.8)	3 (15.7)	2 (22.2)	1.000
K57	4 (14.3)	4 (21)	0 (0.0)	0.303
Virulence factors				
Hypermucoviscosity-related gene				
*rmpA*	2 (7.1)	0 (0.0)	2 (22.2)	0.126
*rmpA2*	2 (7.1)	0 (0.0)	2 (22.2)	0.126
CPS synthesis				
*wcaJ*	9 (32.1)	0 (0.0)	9 (100)	<0.001^*a*^
Lipopolysaccharide synthesis				
*wabG*	26 (92.8)	17 (89.4)	9 (100)	1.000
*uge*	22 (78.5)	13 (68.4)	9 (100)	0.564
Adhesin				
*fimH*	22 (78.5)	13 (68.4)	9 (100)	0.564
*mrkD*	23 (82.1)	14 (73.6)	9 (100)	0.769
Iron uptake and transport				
*entB*	22 (78.5)	13 (68.4)	9 (100)	0.564
*ybtS*	11 (39.2)	2 (10.5)	9 (100)	0.010^*a*^
*kfuBC*	25 (89.2)	16 (84.2)	9 (100)	0.780
*iucA*	2 (7.1)	0 (0.0)	2 (22.2)	0.126
*iutA*	2 (7.1)	0 (0.0)	2 (22.2)	0.126
*iroN*	2 (7.1)	0 (0.0)	2 (22.2)	0.126
Allantoin regulon				
*allS*	8 (28.5)	4 (21)	4 (44.4)	0.421
pLVPK-associated markers				
*rmpA*, *rmpA2*, *iroN*, *iucA*, and *iutA*	2 (7.1)	0 (0.0)	2 (22.2)	0.126

a *P* values that denote statistical significance.

b CS-hvKP, carbapenem-susceptible hvKP; CR-hvKP, carbapenem-resistant hvKP.

### AMR determinants.

Various β-lactamase genes (ESBLs, AmpCs, and carbapenemases), aminoglycoside resistance genes, and quinolone resistance genes were identified in hvKPs (Table 1).

**FIG 1:**
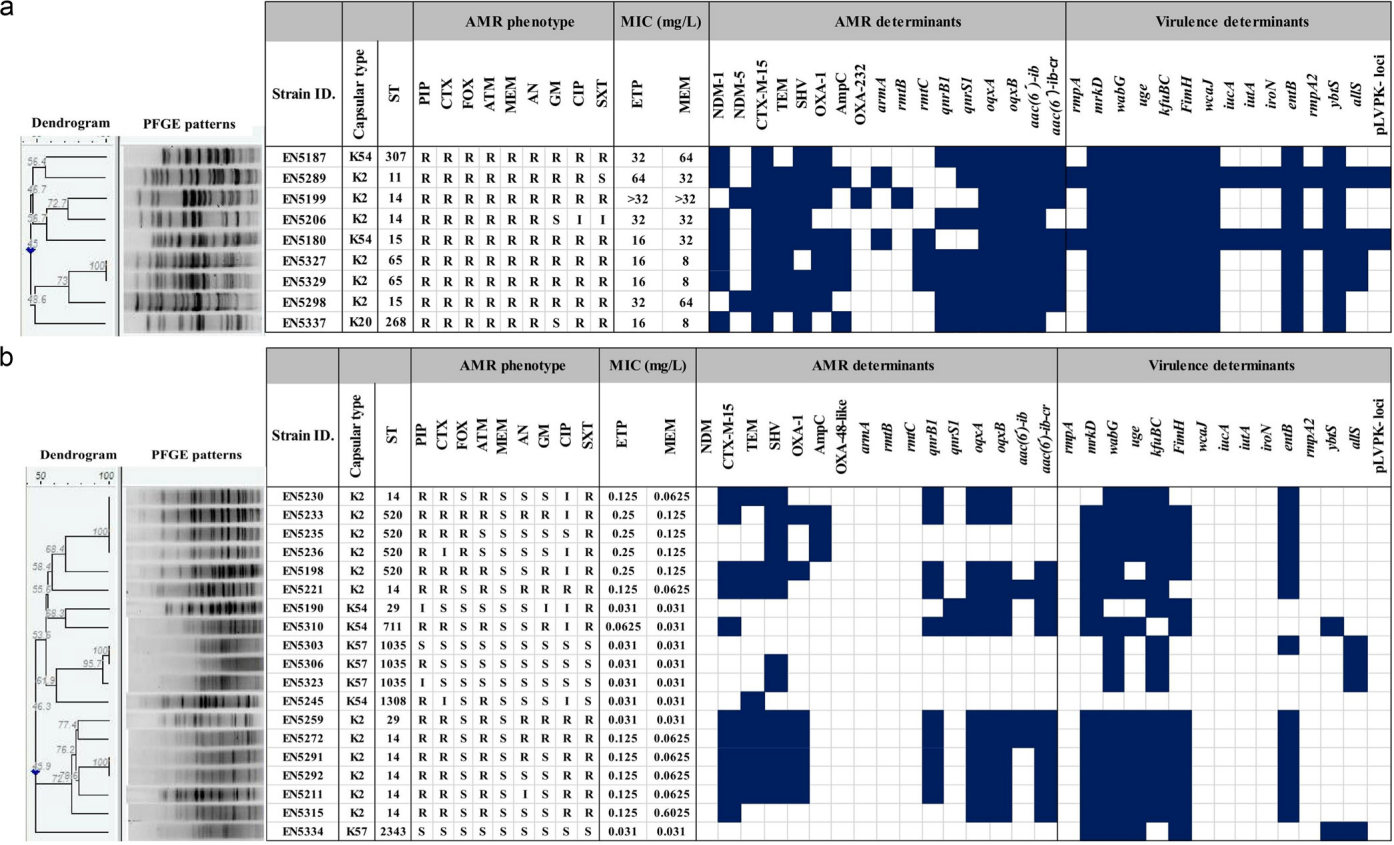
PFGE profiles along with distribution of different antimicrobial resistance (both phenotypic and genotypic) and virulence determinants of the neonatal septicemic, carbapenem-resistant (a) and carbapenem-susceptible (b) hvKP strains. Dark blue filled-up boxes indicate the presence of different antimicrobial resistance and virulence determinants.

Moreover, out of 9 CR-hvKPs, 7 harbored *bla*_NDM-1_, and 2 harbored *bla*_NDM-5_. One strain co-harbored *bla*_NDM-5_ and *bla*_OXA-232_. Among the several AMR determinants, *rmtC* (*P = *0.048), *aac(6′)-ib* (*P = *0.017), and *qnrS1* (*P = *0.023) were significantly higher in CR-hvKPs (Table 1). Figure 1 depicts the distribution of AMR determinants.

### Virulence-associated analysis.

K2 (64.2%, 18/28) was the most prevalent of the identified capsular types, followed by K54 (17.8%, 5/28) and K57 (14.3%, 4/28) (Table 1). Moreover, K2 and K54 were detected in both carbapenem-resistant and carbapenem-susceptible strains. Several virulence determinants, *viz.*, *wabG*, *kfuBC*, *mrkD*, *fimH*, *entB*, *uge*, *ybtS*, and *wcaJ* were identified in hvKPs; of them, *wcaJ* (*P < *0.001) and *ybtS* (*P = *0.010) were significantly higher in CR-hvKPs (Table 1). In contrast, pLVPK-associated markers (*rmpA*, *rmpA2*, *iroN*, *iucA*, and *iutA*) were detected only in 2 NDM-1-producing CR-hvKP strains (EN5180 and EN5289). Figure 1 depicts the distribution of various virulence factors.

Additionally, 42.8% (12/28) of strains possessed strong biofilm-forming ability (OD_595_ = 0.82 to 1.23) compared to the negative control (OD_595_ = 0.27), and 78.5% (22/28) of strains exhibited high level of serum resistance (100% viable counts after 3 h of incubation) compared to the positive control. Table S1 summarizes *in vitro* virulence results.

### Clonal relatedness among hvKP strains.

Pulsed-field gel electrophoresis (PFGE) revealed that most of the studied hvKPs (60.7%, 17/28) were clonally diverse. However, among the 9 CR-hvKPs, EN5327 and EN5329 were clonal, and among the 19 carbapenem-susceptible strains, 3 distinct clonal-clusters were observed (Cluster I – EN5230, EN5233, EN5235, and EN5236; Cluster II – EN5303, EN5306, and EN5323; Cluster III – EN5291 and EN5292), while the rest were distinct (Fig. 1).

The hvKPs consisted of 12 different STs (ST11, ST14, ST15, ST29, ST65, ST268, ST307, ST520, ST711, ST1035, ST1308, and ST2343) (Fig. 1). Of them, ST14 was the most common (32.1%, 9/28), and was associated with both carbapenem-resistant and carbapenem-susceptible populations. Additionally, ST11, ST15, ST65, ST268, and ST307 were associated only with CR-hvKPs. Other STs (ST29, ST520, ST711, ST1035, ST1308, and ST2343) were detected only in carbapenem-susceptible strains.

### Clinical outcome of the neonates infected with hvKP strains.

Table 2 represents the clinical status of the hvKP-infected neonates. Out of 28 neonates, 16 (57.1%) were outborn (refers to the neonates referred from some other facility or extramural births), and most were delivered preterm (53.5%, 15/28), were of low birth weight (57.1%, 16/28), or extremely low birth weight (17.8%, 5/28). 39.2% (11/28) of neonates who succumbed to the condition, suffered from severe clinical complications, as listed in Table 2. Notably, 33.3% (6/18) of neonates infected with hvKP-K2 strains did not survive.

**TABLE 2:** Clinical and demographic characteristics of the septicemic neonates (*n* = 28) infected with the hvKP and/or CR-hvKP strains

Strain No.	Capsular type(s)	ST^*a*^(s)	Time of isolation	Clinical complications	Sex	Inborn^*b*^/outborn^*c*^	Birth wt (g)	Mode of delivery	Gestational age (wk)		Outcome
Neonates infected with carbapenem-resistant hvKP (*n* = 9)										
EN5180	K54	15	April, 2014	Sepsis, perinatal asphyxia, respiratory distress	M^*d*^	Inborn	1,400	LUCS^*f*^	34		Expired
EN5187	K54	307	June, 2014	Hyperglycemia, apnea, sepsis, and meningitis	M	Outborn	995	NVD^*g*^	31		Expired
EN5199	K2	14	November, 2014	Gastroschisis	F^*e*^	Inborn	2,223	NVD	36		Expired
EN5206	K2	14	February, 2015	Septic arthritis, perinatal asphyxia, HIE-II^*h*^, intracranial hemorrhage	M	Outborn	2,250	NVD	37		Expired
EN5289	K2	11	March, 2016	Agonal breathing, ELBW^*i*^, IVH-III^*j*^, septic shock	M	Outborn	650	NVD	30		Expired
EN5298	K2	15	April, 2016	Respiratory distress, ELBW, sepsis, apnea	F	Outborn	835	NVD	28		Discharged
EN5327	K2	65	June, 2016	Bilious vomiting, intestinal obstruction	M	Outborn	1,640	NVD	34		Expired
EN5329	K2	65	July, 2016	Hyperbilirubinemia	F	Inborn	2,420	NVD	35		Discharged
EN5337	K20	268	October, 2016	Perinatal asphyxia, HIE-II	M	Outborn	2,700	NVD	37		Discharged
Neonates infected with carbapenem-susceptible hvKP (*n* = 19)										
EN5190	K54	29	August, 2014	Born with MSL^*k*^, hematuria	F	Inborn	2,118	LUCS	37		Discharged
EN5198	K2	520	November, 2014	Respiratory distress, BCA^*l*^	F	Outborn	2,400	LUCS	37		Expired
EN5211	K2	14	April, 2015	IUGR^*m*^, hyperbilirubinemia, intestinal perforation, culture-positive sepsis, septic shock, DIC^*n*^	F	Inborn	850	NVD	35		Expired
EN5221	K2	14	August, 2015	LBW^*o*^, sepsis	F	Outborn	1,900	NVD	37		Discharged
EN5230	K2	14	September, 2015	Perinatal asphyxia, HIE-II	M	Inborn	3,876	NVD	41		Discharged
EN5233	K2	520	September, 2015	Respiratory distress syndrome	M	Inborn	2,164	LUCS	35		Discharged
EN5235	K2	520	September, 2015	Rh hemolytic disease, hyperbilirubinemia	M	Inborn	2,784	LUCS	38		Discharged
EN5236	K2	520	September, 2015	PR^*p*^ Bleeding	F	Inborn	2,807	LUCS	37		Discharged
EN5245	K54	1308	October, 2015	Hyperbilirubinemia	M	Inborn	1,971	LUCS	36		Discharged
EN5259	K2	29	November, 2015	Poor feeding	M	Outborn	3,006	NVD	37		Discharged
EN5272	K2	14	December, 2015	Preterm^*q*^	F	Outborn	2,260	NVD	27		Discharged
EN5291	K2	14	March, 2016	Delayed passage of meconium, Hirschsprung’s disease	M	Outborn	1,900	NVD	35		Discharged
EN5292	K2	14	March, 2016	Sepsis, DIC, GDM^*r*^	F	Outborn	1,550	LUCS	37		Discharged
EN5303	K57	1035	April, 2016	ELBW, pulmonary hemorrhage	M	Outborn	945	NVD	29		Expired
EN5306	K57	1035	April, 2016	Jitteriness, hypoglycemia, hypocalcemia, septic shock	F	Inborn	2,124	NVD	36		Expired
EN5310	K54	711	May, 2016	Abdominal distension, intestinal obstruction	M	Outborn	2,250	NVD	38		Expired
EN5315	K2	14	May, 2016	Abdominal distension	M	Outborn	3,074	NVD	40		Discharged
EN5323	K57	1035	June, 2016	Respiratory distress	M	Outborn	2,700	LUCS	39		Discharged
EN5334	K57	2343	September, 2016	Respiratory distress, esophageal atresia, cleft lip and palate	F	Inborn	1,803	LUCS	36		Discharged

a ST, sequence type.

b Inborn, refers to the neonates born at the tertiary care hospital (intramural birth).

c Outborn, refers to the neonates referred from some other facility (extramural birth).

d M, male.

e F, female.

f LUCS, lower uterine segment cesarean section.

g NVD, normal vaginal delivery.

h HIE-II, hypoxic-ischemic encephalopathy grade II.

i ELBW, extremely-low-birth weight (<1,000 g).

j IVH-III, intraventricular hemorrhage grade III.

k MSL, meconium-stained liquor.

l BCA, bilateral choanal atresia.

m IUGR, intrauterine growth restriction.

n DIC, disseminated intravascular coagulation.

o LBW, low birth weight (<2,500 g).

p PR bleeding, per rectal bleeding.

q Preterm, <37 weeks of gestational age.

r GDM, gestational diabetes mellitus.

### Analysis of transconjugants.

Conjugal transfer of *bla*_NDM_ was successful in all cases. All transconjugants exhibited MIC for carbapenems ranging between 2 and 32 mg/L. However, Tc-EN5199 with dual carbapenemases (*bla*_NDM-5_ and *bla*_OXA-232_) showed higher MIC for ertapenem and meropenem (Table 3). *bla*_NDM-1_/*bla*_NDM-5_, along with several other AMR determinants, was borne on different conjugative plasmid scaffolds, *viz.*, IncA/C, IncR, IncFII, IncFIIK, and IncHIB-M/FIB-M (Table 3).

**TABLE 3:** Microbiological characteristics of the *bla*_NDM_-harboring parental strains and their transconjugants

Strains	ST^*a*^	MIC (mg/L)		AMR determinants	pLVPK^*d*^-associated markers	Inc group
		ETP^*b*^	MEM^*c*^			
EN5180^*f*^	15	16	32	*bla*_NDM-1_, *bla*_CTX-M-15_, *bla*_TEM-1_, *bla*_SHV_, *bla*_OXA_, *bla*_CMY-6_, *armA*, *rmtC*, *oqxAB*, *aac(6′)-ib*, *aac(6′)-ib-cr*	*rmpA*, *rmpA2*, *iucA*, *iutA*, *iroN*	A/C, FIIK, HIB-M, FIB-M
Tc^*e*^-EN5180^*f*^	-^*g*^	4	8	*bla*_NDM-1_, *bla*_TEM-1_, *rmtC*, *aac(6′)-ib*	-	A/C
EN5187	307	32	64	*bla*_NDM-1_, *bla*_CTX-M-15_, *bla*_SHV_, *bla*_OXA_, *qnrB1*, *qnrS1*, *oqxAB*, *aac(6′)-ib*, *aac(6′)-ib-cr*	-	FIIK, L/M, FII
Tc-EN5187	-	8	8	*bla*_NDM-1_, *bla*_CTX-M-15_, *qnrB1*, *qnrS1*, *aac(6′)-ib*, *aac(6′)-ib-cr*	-	FIIK
EN5199	14	>32	>32	*bla*_NDM-5_, *bla*_OXA-232_, *bla*_CTX-M-15_, *bla*_TEM-1_, *bla*_SHV_, *bla*_OXA_, *rmtB*, *oqxAB*, *aac(6′)-ib*, *aac(6′)-ib-cr*	-	R, FIIK, FII, FIB, FIA, Col
Tc-EN5199	-	32	24	*bla*_NDM-5_, *bla*_OXA-232_, *bla*_CTX-M-15_, *bla*_TEM-1_, *rmtB*	-	R, FIIK, Col
EN5206	14	32	32	*bla*_NDM-1_, *bla*_CTX-M-15_, *bla*_TEM-1_, *bla*_SHV_, *qnrB1*, *qnrS1*, *oqxAB*, *aac(6′)-ib*	-	FII, FIIK, R
Tc-EN5206	-	8	4	*bla*_NDM-1_, *bla*_CTX-M-15_, *qnrS1*, *aac(6′)-ib*	-	FIIK
EN5289^*f*^	11	64	32	*bla*_NDM-1_, *bla*_CTX-M-15_, *bla*_TEM-1_, *bla*_SHV_, *bla*_OXA,_ *bla*_DHA-1_, *armA*, *qnrS1*, *oqxAB*, *aac(6′)-ib*, *aac(6′)-ib-cr*	*rmpA*, *rmpA2*, *iucA*, *iutA*, *iroN*	FIIK, HIB-M, FIB-M, HI1
Tc-EN5289^*f*^	-	8	4	*bla*_NDM-1_, *bla*_CTX-M-15_	*rmpA*, *rmpA2*, *iucA*, *iutA*, *iroN*	HIB-M, FIB-M
EN5298	15	32	64	*bla*_NDM-5_, *bla*_CTX-M-15_, *bla*_TEM-1_, *bla*_SHV_, *bla*_OXA_, *qnrB1*, *qnrS1*, *oqxAB*, *aac(6′)-ib*, *aac(6′)-ib-cr*	-	FIIK, FIB, FIA
Tc-EN5298	-	4	2	*bla*_NDM-5_, *bla*_CTX-M-15_, *qnrS1*, *aac(6′)-ib*, *aac(6′)-ib-cr*	-	FIIK
EN5327	65	16	8	*bla*_NDM-1_, *bla*_CTX-M-15_, *bla*_TEM-1_, *bla*_OXA_, *bla*_ACT-69_, *rmtC*, *qnrB1*, *qnrS1*, *oqxAB*, *aac(6′)-ib*, *aac(6′)-ib-cr*	-	FII, FIIK, X3
Tc-EN5327	-	8	4	*bla*_NDM-1_, *bla*_CTX-M-15_, *bla*_TEM-1_, *rmtC*, *qnrB1, aac(6′)-ib*, *aac(6′)-ib-cr*	-	FII
EN5329	65	16	8	*bla*_NDM-1_, *bla*_CTX-M-15_, *bla*_TEM-1_, *bla*_SHV_, *bla*_OXA_, *bla*_ACT-69_, *rmtC*, *qnrB1*, *qnrS1*, *oqxAB*, *aac(6′)-ib*, *aac(6′)-ib-cr*	-	FII, FIIK, X3
Tc-EN5329	-	8	4	*bla*_NDM-1_, *bla*_CTX-M-15_, *bla*_TEM-1_, *rmtC*, *qnrB1, aac(6′)-ib*, *aac(6′)-ib-cr*	-	FII
EN5337	268	16	8	*bla*_NDM-1_, *bla*_CTX-M-15_, *bla*_SHV_, *bla*_DHA-1_, *qnrB1*, *qnrS1*, *oqxAB*, *aac(6′)-ib*	-	R, FIIK, HIB-M, FIB-M
Tc-EN5337	-	4	2	*bla*_NDM-1_, *bla*_CTX-M-15_, *qnrB1*, *qnrS1*, *aac(6′)-ib*	-	R, FIIK

a ST, sequence type.

b ETP, ertapenem.

c MEM, meropenem.

d pLVPK, large virulence plasmid of K. pneumoniae.

e Tc, E. coli J53 transconjugants selected in LB agar medium containing 1 mg/L ertapenem and 100 mg/L sodium azide.

f pLVPK-markers-harboring CR-hvKP strains and their respective Tc.

g -, absence of any particular attribute.

Moreover, among the identified transconjugants, only Tc-EN5289 tested positive for *bla*_NDM-1_, *bla*_CTX-M-15_, pLVPK-associated markers, and harbored a conjugative IncHIB-M/FIB-M plasmid. This co-transfer of pLVPK-markers along with *bla*_NDM-1_ suggests the possible association of both highly transmissible AMR and hypervirulence determinants in the same conjugative plasmid (Table 3).

Except for a difference in a few AMR and virulence determinants, no other significant difference was observed between the carbapenem-resistant and carbapenem-susceptible K. pneumoniae in terms of *in vitro* virulence assessment and demographics of the neonates.

### Resistome, virulome, and comparative genomic analysis of CR-hvKP EN5180 and EN5289 strains.

The genomes of EN5180 (~ 6.1 Mb, 56.43% G+C, 5974 CDS) and EN5289 (5.9 Mb, 56.68% G+C, 5902 CDS) harbored a plethora of different AMR determinants, several plasmid replicons, heavy-metal resistance determinants, and efflux pumps and regulator elements (Table 4).

**TABLE 4:** Genome-level characteristics of neonatal septicemic CR-hvKP strains EN5180 and EN5289

Variable	Strain information	
Strain name	K. pneumoniae EN5180	K. pneumoniae EN5289
Sequence type	ST15	ST11
Capsular type	K54	K2
Genomic features		
Genome size (bp)	6,131,075	5,967,685
Contig no.	115	107
GC content (%)	56.43	56.68
CDS^*a*^	5,974	5,902
rRNA genes	14	34
tRNA genes	74	80
tmRNA^*b*^ genes	1	1
ncRNA^*c*^	13	11
Pseudogenes	146	135
Repeat region	3	1
Antibiotic resistance genes		
Aminoglycoside resistance	*aac(6′)-Ib3*, *aadA1*, *armA*, *rmtC*	*aac(6′)-Ib*, *aadA1*, *armA*
β-lactam resistance	*bla*_SHV-28_, *bla*_SHV-106,_ *bla*_TEM-1A_, *bla*_OXA-9_, *bla*_CTX-M-15_, *bla*_CMY-6_, *bla*_NDM-1_	*bla*_SHV-182_, *bla*_TEM-1B_, *bla*_OXA-9_, *bla*_CTX-M-15_, *bla*_DHA-1_, *bla*_LAP-2_, *bla*_NDM-1_
Quinolone resistance	*aac(6′)-Ib-cr*, *oqxA*, *oqxB*	*aac(6′)-Ib-cr*, *oqxA*, *oqxB*, *qnrS1*
Fosfomycin resistance	*fosA*	*fosA*
Macrolide resistance	*msr*(E)	*msr*(E)
Sulphonamide resistance	*sul1*	*sul1*
Trimethoprim resistance	*-* ^*f*^	*tet(A)*
Plasmid types		
Col plasmids	Col440I	Col440I, Col(BS512)
F plasmids	IncFIB(pKPHS1), IncFIB(pNDM-Mar), IncFIB(pQil), IncFII(K)	IncFIB(pNDM-Mar), IncFIB(pQil), IncFII(K)
Other plasmids	IncHI1B(pNDM-MAR), IncA/C2	IncHI1B(pNDM-MAR)
Heavy metal resistance genes		
Silver resistance	*silCERS*	*silCERS*
Tellurite resistance	*terABCDEWXYZ*	*terABCDEWXYZ*
Efflux pumps and regulators		
RND^*d*^ family efflux pump	*acrAB*	*acrAB, mexB, mdtL*
HTH^*e*^ type transcriptional regulator	*acrR*	*acrR*
AraC/XylS family transcriptional activator	*marAB*, *soxRS*, *rob*, *ramA*, *rarA*	*marAB*, *soxRS*, *rob*, *ramA*, *rarA*
TetR family transcriptional regulator	*ramR*	*ramR*
HTH LuxR type transcriptional regulator	*sdiA*	*sdiA*
rRNA transcriptional activator	*fis*	*fis*
Suppressor of efflux pump	*envR*	*envR*
Quinolone and Olaquindox efflux pump	*oqxABR*	*oqxABR*
Virulence genes		
Type 3 fimbriae	*mrkABCDFHIJ*	*mrkABCDFHIJ*
Type 1 fimbriae	*fimABCDEFGHIK*	*fimABCDEFGHIK*
Type IV pili biosynthesis	*-*	*pilU*
ABC iron transporter	*kfuABC*	*kfuABC*
Aerobactin	*iucABCD*; *iutA*	*iucABCD*; *iutA*
Enterobactin	*entABCDEFS*; *fepABCDG*; *fes*	*entABCDEFS*; *fepABCDG*; *fes*
Salmochelin	*iroBCDEN*	*iroBCDEN*
Yersiniabactin	*fyuA*; *irp1, irp2*; *ybtAEPQSTUX*	*fyuA*; *irp1, irp2*; *ybtAEPQSTUX*
Allantoin utilization	*allABCDRS*	*allABCDRS*
Capsule regulators	*rcsAB*; *rmpA*, *rmpA2*	*rcsAB*; *rmpA*, *rmpA2*
pLVPK-derived loci	*rmpA*, *rmpA2*; *iroBCDEN*; *iucABCDiutA*; *peg-344*	*rmpA*, *rmpA2*; *iroBCDEN*; *iucABCDiutA*; *peg-344*
Secretion system	T6SS (I–III)	T6SS (I–III)
Other factors (Autotransporter, Iron/manganese uptake, and Stress adaptation)	*flu*; *sitABCD*; *mntB*	-
BioProject no.	PRJNA684006	
Accession no.	JAELUV000000000	JAELUW000000000

a CDS, coding sequence.

b tmRNA, transfer-mRNA.

c ncRNA, non-coding RNA.

d RND, resistance-nodulation-division.

e HTH, helix-turn-helix.

f -, absence of any particular attribute.

Moreover, EN5180 and EN5289 harbored a diverse array of putative virulence factors, including gene clusters for type 1 and type 3 fimbriae, ABC iron-transporter, aerobactin, enterobactin, salmochelin, yersiniabactin, allantoin utilization, capsule regulators, and multiple type-VI secretion systems (T6SS I - III) (Table 4). WGS confirmed the presence of several pLVPK-associated markers (*rmpA*, *rmpA2*, *iroBCDEN*, *iucABCDiutA*, and *peg-344*) in both the strains (Table 4), which were missing in the other hvKP strains. An alignment of EN5180 and EN5289 with pLVPK (GenBank accession AY378100) demonstrated the existence of pLVPK-like sequence in the studied genomes (Fig. S2). Furthermore, EN5180 and EN5289 possessed several prophage elements (Table S2 and Fig. S3) and CRISPR arrays (Fig. S4).

**FIG 2:**
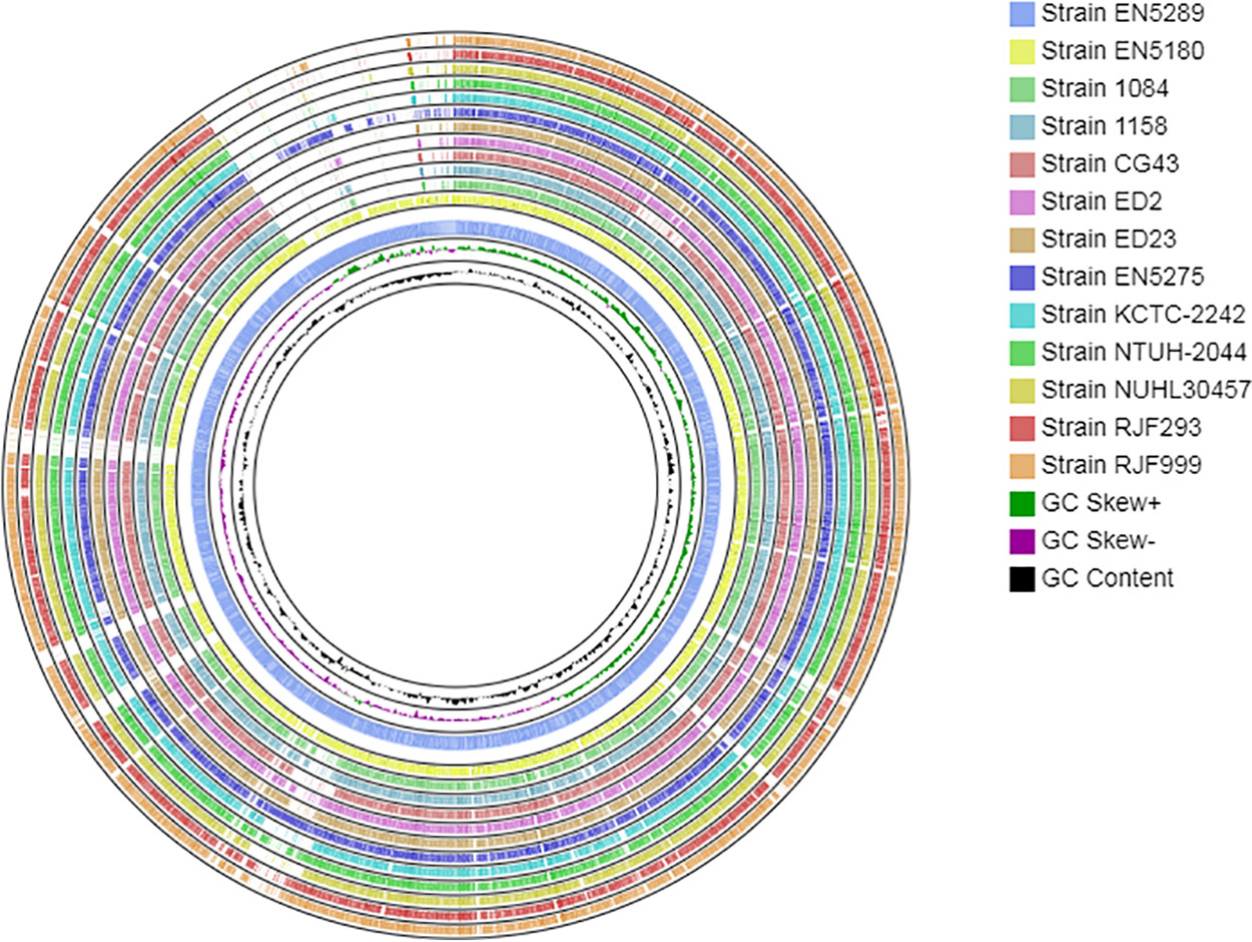
Comparative BLASTN analysis of NDM-1-producing neonatal septicemic CR-hvKP strains EN5180 and EN5289 and 11 other hvKP reference strains. The circular map was generated using the CGView^BETA^ comparison tool. From the center to the periphery: Ring 1 and 2 show the GC content and GC skew, respectively; Ring 3 and ring 4 display CR-hvKP strains EN5289 (accession: JAELUW000000000) and EN5180 (accession: JAELUV000000000), respectively; Ring 4: strain 1084 (accession: CP003785.1); Ring 5: strain 1158 (accession: CP006722.1); Ring 6: strain CG43 (accession: CP006648.1); Ring 7: strain ED2 (accession: CP016813.1); Ring 8: strain ED23 (accession: CP016814.1); Ring 9: strain EN5275 (accession: VINI00000000); Ring 10: strain KCTC-2242 (accession: CP002910.1); Ring 11: strain NTUH-2044 (accession: NC_012731.1); Ring 12: strain NUHL30457 (accession: CP026586.1); Ring 13: strain RJF293 (accession: CP014008.1); Ring 14: strain RJF999 (accession: CP014010.1). The sequence comparison revealed that the 13 hvKP strains were shown to have a high degree of similarity.

Comparative genomic (BLAST+ and BLASTN) analysis of EN5180 and EN5289 with the other hvKP reference genomes revealed high inter-genomic resemblance (*in silico* DNA-DNA hybridization values ~ 94%) (Fig. 2 and Table S3). Phylogenomic analysis also showed that EN5180 and EN5289 were highly similar to the other hvKP genomes (Fig. S5).

**FIG 3:**
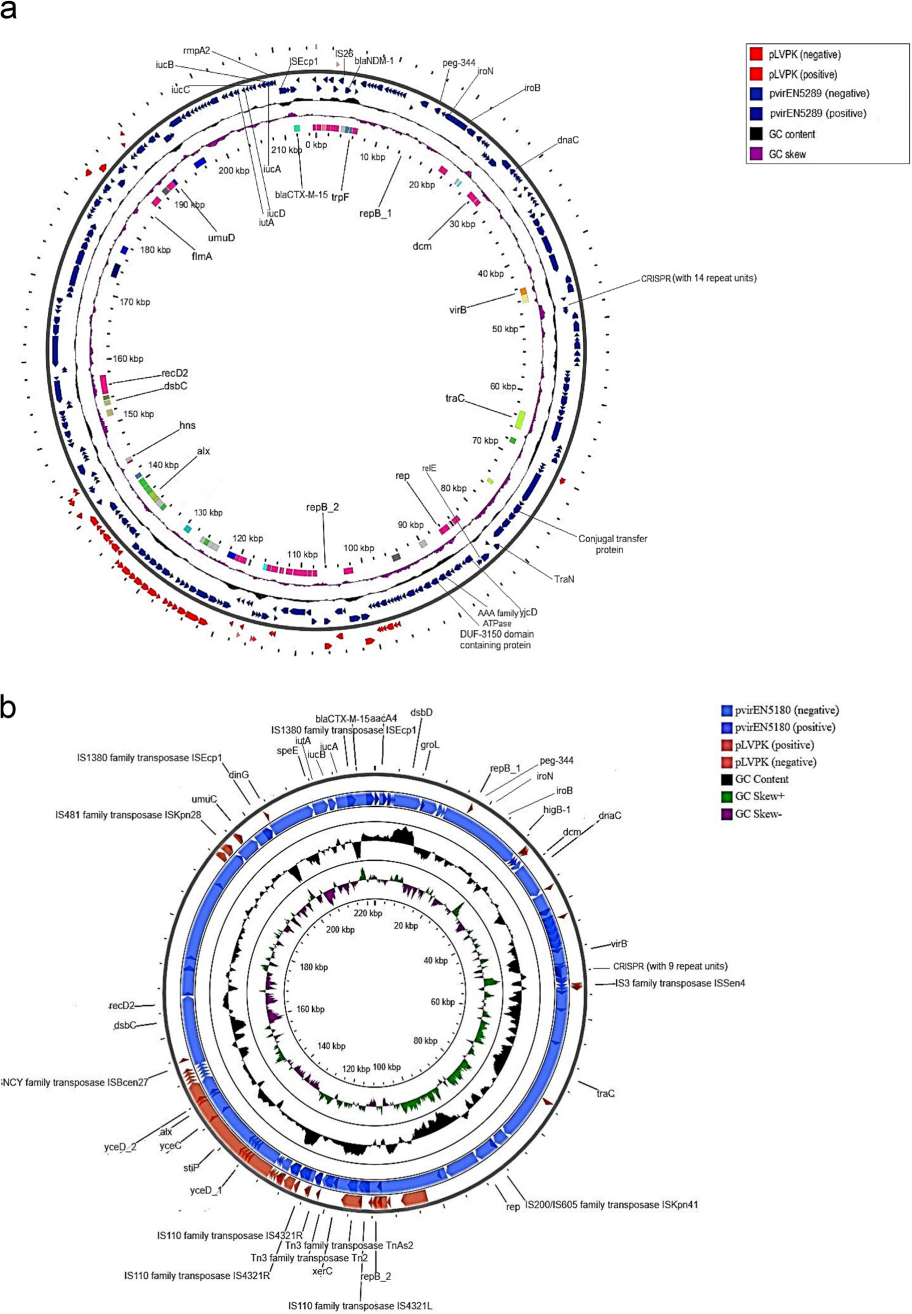
Virulence plasmid sequence alignment analysis among the *bla*_NDM-1_ and hypervirulence determinants co-encoding hybrid plasmid, pvirEN5289, and the previously reported virulence plasmid, pLVPK (GenBank accession AY378100) (a), sequence alignment analysis among the *bla*_CTX-M-15_ and hypervirulent markers co-harboring hybrid plasmid, pvirEN5180 and pLVPK (b). Although, both the genomes harbored all the hypervirulent biomarkers, some of the molecular markers were not portrayed in the given virulence plasmid maps due to their low sequence identity against the available sequence databases.

In addition, *in silico* virulence plasmid (pLVPK-like) analysis of EN5289 revealed that *bla*_NDM-1_ co-existed with several pLVPK-markers (*rmpA2*, *iroB*, *iroN*, *iucABCDiutA*, and *peg-344*) on the same ~ 210 kb IncHI1B/IncFIB plasmid, henceforth designated as pvirEN5289 (Fig. 3a). *bla*_NDM-1_ was associated with IS*26* and *bla*_CTX-M-15_ with IS*Ecp1* (at their upstream). Notably, the presence of type IV secretion system (T4SS) and the conjugal transfer (*tra*) genes in pvirEN5289, firmly substantiates its conjugative nature. The result of the aforementioned analysis in combination with the transconjugant ([Tc]-EN5289) data (Table 3), strongly suggests that EN5289 harbored a conjugative *bla*_NDM-1_, and hypervirulence determinants co-encoding hybrid plasmid, pvirEN5289. Additionally, another ~ 220 kb hybrid IncHI1B/IncFIB plasmid was detected in EN5180, designated as pvirEN5180 (Fig. 3b), which also co-harbored *bla*_CTX-M-15_ and various pLVPK-markers (*iroB*, *iroN*, *iucABiutA*, and *peg-344*). Unlike EN5289, pLVPK-markers and *bla*_NDM-1_ were carried on separate plasmids in EN5180, as *bla*_NDM-1_ was transferred via conjugation but no pLVPK-markers were detected in Tc-EN5180.

Notably, sequence alignments with BLAST revealed that pvirEN5180 and pvirEN5289 shared ~ 82 and ~ 81% query coverage with pLVPK (GenBank accession AY378100), respectively.

### *In vivo* virulence of EN5180 and EN5289.

**FIG 4:**
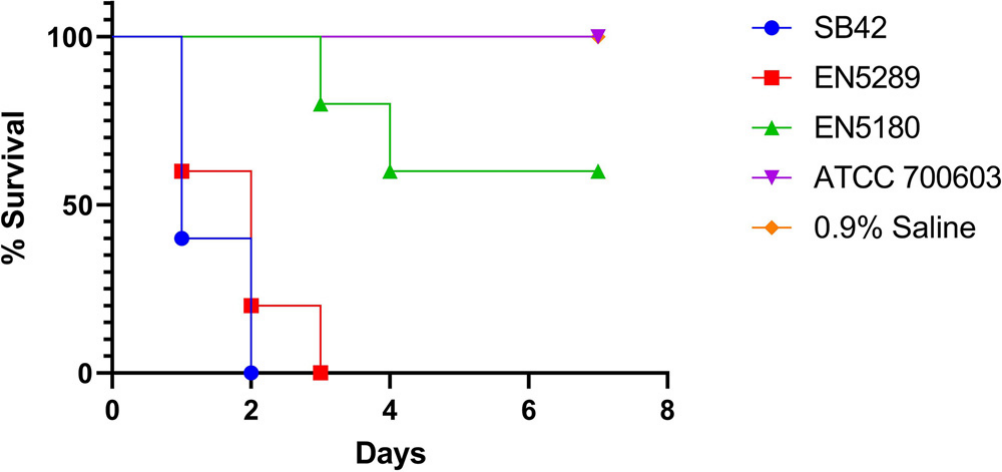
Kaplan–Meier survival curves for neonatal septicemic, carbapenem-resistant hypervirulent K. pneumoniae EN5180- and EN5289-infected mice. Female BALB/c mice (4 to 6-weeks-old, 5 mice/strain) were intraperitoneally challenged with 1 × 10^5^ CFU of the different K. pneumoniae strains (EN5180, EN5289, hypervirulent control strain SB42, and low virulence control strain ATCC 700603). Saline was administered as vehicle control. Mice were monitored every 24 h up to 7 days, and scored for death. During the 7-day period, no mice in the ATCC 700603 or saline groups died.

The Kaplan-Meier survival analysis revealed that mice infected with EN5289 portrayed virulence similar to that of the hvKP-K1 positive control strain SB42 (Fig. 4), indicating that EN5289 was equivalently virulent to the well-established hvKP-K1 strain in this model. However, despite harboring all the hypervirulent genetic traits, higher inoculum (10^5^ CFU) of EN5180 did not lead to significant mouse lethality.

## DISCUSSION

The recent emergence of antibiotic-resistant hvKP is a serious public-health concern (15). Due to the absence of awareness about hvKP and the lack of a universally agreed consensus definition/marker for hypervirulence, clinical microbiology laboratories are unable to differentiate hvKP from cKP in routine diagnosis, potentially affecting the treatment regimen (18, 19).

Several recent studies have shown that K. pneumoniae is the major cause of neonatal sepsis (2); however, such studies lack a separate investigation regarding hvKPs. Reports of neonatal hvKP cases are just a handful (19), and there are no studies where hvKP strains causing neonatal sepsis have been characterized in terms of resistance and virulence over an extended period of time. To understand hvKP in a new and vulnerable epidemiological context, this study was carried out.

To date, there is no consensus on the definition of hvKP. Early studies have differentiated hvKP by the severity of clinical representation or hypermucoviscous phenotype, and later by capsular/sequence types or presence of certain plasmid-borne virulence markers (14). A consensus is required to interpret characteristics (cKP or hvKP) and compare strains globally (18). The characterization of neonatal strains here endeavors to fill some gaps in this knowledge. Considering the immune-evasive and severe invasive disease-causing ability of some capsular types (K1, K2, K5, K20, K54, and K57) (14), hvKP strains in this study were selected based on the above-stated capsular types.

Nearly 26% of the neonatal septicemic strains were categorized as hvKP based on the capsular types. As there are no reports of prevalence of hvKP in neonatal population, we compared our result with the hvKP cases causing infections in adult population. Our findings revealed that the incidence of hvKP was lower than those reported from China (33%), Taiwan (38%), and Korea (42.4%) (20–22), but higher than the cases reported from Spain (5.4%) and Canada (8.2%) (23, 24). This differential prevalence of hvKP might be partly attributed to the geographical and/or demographical differences, and the characteristics selected to define hvKP strains.

Majority of the studied hvKPs (64.2%, 18/28) belonged to capsular type K2, which also resulted in 33.3% (6/18) of neonatal mortality. The occurrence of K2 in hvKP cases were reported from China (42.9% to 68.7%) (25, 26). However, a large proportion of studies also isolated K1 strains from China and Taiwan, but no K1 serotype was identified from this setup, except one septicemic CR-hvKP ST23 (OXA-232-producing) strain that has been reported earlier (17).

The studied hvKP strains were diverse, belonging to 12 different STs, some of which (ST11, ST14, ST15, ST29, ST65, ST268) have recently been detected in several other hvKP cases (15, 16, 27). The diversity of clones in the study could be from different exposures, as most hvKP-infected neonates were referred from other hospitals with exposure to different nosocomial and/or environmental factors. Previous studies have noted that hvKP-K1 strains belong predominantly to clonal cluster 23 (CC23), and can be grouped into a distinct monophyletic clade. In contrast, hvKP-K2 strains belong to genetically unrelated groups. The diversity of the studied strains is consistent with these reports (28). Such convergence of hvKP in diverse STs indicates a plausible clonal expansion of the pathotype, which is worrisome.

In this study, carbapenem resistance in CR-hvKPs was primarily conferred by *bla*_NDM_. One strain with dual carbapenemases (*bla*_NDM-5_ and *bla*_OXA-232_) was also detected. The presence of *bla*_NDM,_ on a variety of conjugative plasmids, suggests the promiscuous nature of this gene (5). To date, most of the reported CR-hvKPs were found to be associated with *bla*_OXA-48-like_ and/or *bla*_KPC-2_ (15–17); however, our study, in concordance with the other recent reports, demonstrated that the NDM-1-producing K. pneumoniae are also gradually becoming hypervirulent (29, 30), making the clinical scenario complicated.

hvKP strains often feature several pLVPK-borne markers (*rmpA*, *rmpA2*, *iro*, *iuc*, and/or *peg-344*) (12), which are essential for the hypervirulent phenotype. Of the 28 hvKPs, only 2 NDM-1-producing strains (EN5180 and EN5289) possessed such markers. Apart from harboring diverse AMR determinants, EN5180 and EN5289 were equipped with a wide range of virulence/hypervirulence determinants, and exhibited strong biofilm-forming and high serum resistance abilities. We speculate that these determinants probably enabled them to persist in the antibiotic-laden nosocomial environments, and also elicit colonization within the hosts (31). Although EN5180- and EN5289-infected neonates succumbed to the condition, the mouse lethality assay carried out in this study suggested that only EN5289 was equivalently virulent to the hvKP-K1 strain SB42. The severity of illness (very low birth weight, prematurity, and several clinical complications) in the neonate infected with EN5180, could be a reason for the fatal outcome. The presence of hypervirulent markers (*rmpA*, *rmpA2*, *iroBCDEN*, *iucABCDiutA*, and *peg-344*), pLVPK-like sequence, pLVPK-associated replicon type (IncHI1B/IncFIB), high inter-genomic resemblance with the other hvKP genomes, both *in vitro* and *in vivo* virulence potential, and fatal clinical outcome confirmed the hypervirulence of the EN5180 ST15-K54 and EN5289 ST11-K2 strains.

Most hvKP strains were, until recently, susceptible to various antimicrobials. Strains, such as ST11 and ST15, which are well-known international high-risk CRKP clones causing several hospital-mediated outbreaks, previously lacked the hypervirulence genes. However, studies suggested penetration of virulence plasmids into these clones (15). To date, there are no reports of hvKP/CR-hvKP ST11 and ST15 strains causing neonatal sepsis. Hence, to the best of our knowledge, this study is the first to describe a detailed microbiological, molecular, and genome-level characterization of the NDM-1-producing CR-hvKP ST11-K2 and ST15-K54 strains causing fatal neonatal bloodstream infections. Notably, EN5180 was recovered in 2014, implying that NDM-1-producing CR-hvKP strains might have emerged even before the initial description of *bla*_KPC-2_-harboring CR-hvKP strains, reflecting the importance of this study (32).

Moreover, as noted in recent studies (33), a new form of molecular convergence is also evident in the studied EN5289 ST11-K2 strain harboring a conjugative *bla*_NDM-1_ and hypervirulence determinants co-encoding hybrid plasmid. Co-transmission of such newly-emerged plasmids could directly convert a cKP into CR-hvKP, leading to escalation in prevalence of CR-hvKPs in clinical settings. Recently, in a review, we discussed how CRKP limits the already available treatment options for neonates (19), where the occurrence of CR-hvKP compounds the problem.

Given the looming threat of hvKP/CR-hvKP and the severity of disease associated with it, several preclinical studies have recently suggested various potential strategies to combat the hvKP infections either via the development of capsular polysaccharide-based vaccine and/or anti-capsular monoclonal antibody-based immunotherapy (34). The epidemiological landscape of hvKP is, thus, important for the implementation of these therapeutic approaches. Moreover, apart from the therapeutic perspective, a consensus about the definition, the detection of hvKP by a cost-effective method, understanding the evolution of CR-hvKP, and the possible modes of acquisition of such strains in the neonates need attention. Our study provides a glimpse of the prevalence of hvKPs in a given neonatal setup. Further, multi-centric surveillance studies combining clinical and microbiological features, are needed to better understand the transmission mechanisms and to prevent these strains from further propagating in hospital settings.

## MATERIALS AND METHODS

### Ethics.

This study was approved by the Institutional Ethics Committee of the ICMR-National Institute of Cholera and Enteric Diseases (ICMR-NICED) (No. A-1/2016-IEC). Patient data were anonymized and deidentified prior to analysis. Animal experiments were approved by the Institutional Animal Ethical Committee of the ICMR-NICED (No. NICED/CPCSEA/68/GO/(25/294)/2019-IAEC/SB/1).

### Bacterial strains.

During 2014 to 2016, among a total of 285 culture-positive cases, 107 (2014, *n* = 19; 2015, *n* = 42; 2016, *n* = 46) non-duplicate K. pneumoniae were recovered from blood of septicemic neonates admitted to the level III unit (Neonatal intensive care unit) of an Indian tertiary care hospital. Blood specimens from the sick neonates were collected at the hospital as part of the routine diagnostic procedure. As a standard protocol, 1 mL of blood for culture was drawn with strict aseptic precautions from a peripheral vein of neonates suspected with sepsis, and inoculated in BD Bactec Peds Plus Vial (Becton Dickinson, MD, USA). Blood culture was performed with the automated Bactec 9050 system (Becton, Dickinson). Gram staining was performed for any culture that flagged positive, and the subculture was carried out on appropriate media based on Gram’s stain: MacConkey agar and 5% sheep blood agar (Difco Laboratories, Detroit, MI, USA) for Gram-negative and Gram-positive isolates, respectively. Bacterial identification and antibiotic susceptibility were performed by Vitek-2 compact system and disk diffusion (as per CLSI guidelines) (35), respectively.

### Experimental procedures.

All 107 K. pneumoniae were screened for capsular types, *viz.*, K1, K2, K5, K20, K54, and K57, considered as frequently encountered hvKP genotypes (36, 37). For the capsular genotyping, a multiplex PCR was performed using the primers as reported previously (37). The PCR amplification consisted of an initial denaturation at 95°C for 10 min, followed by 35 cycles of denaturation at 90°C for 30 s, annealing at 58°C for 90 s, extension at 72°C for 90 s, and a final extension at 72°C for 10 min. Multiplex PCR-generated amplicons were further revalidated by singleplex PCR using the same primers. Strains positive for the capsular types were categorized as hypervirulent, and were analyzed for antibiotic susceptibility, AMR and virulence determinants, clonal relatedness, and *in vitro* virulence. Transmissibility of the carbapenemase gene was also assessed via conjugation. Genome-based and *in vivo* analyses were performed for strains harboring pLVPK-associated markers (*rmpA*, *rmpA2*, *iroN*, *iucA*, and *iutA*).

### Determination of MIC, and detection of antibiotic resistance and virulence determinants.

For hvKPs, carbapenem (ertapenem and meropenem) MICs were evaluated by broth microdilution (38), according to CLSI guidelines (35). Phenotypic detection of carbapenemases was also accomplished (Rosco Diagnostica A/S, Taastrup, Denmark).

PCR and sequencing were performed for the following AMR determinants: β-lactamases (*bla*_CTX-M,TEM,SHV,OXA_), AmpCs (*bla*_MOX,CMY,DHA,ACC,MIR/ACT,FOX_), carbapenemases (*bla*_KPC,SME,IMI,GES,NMC_; *bla*_VIM,IMP,SPM,GIM,SIM,NDM_; *bla*_OXA-48-like_), aminoglycoside resistance genes (*rmt-A*,*B*,*C*,*D*, *armA*, and *aac(6′)-Ib*), and quinolone resistance genes (*qnr-A*,*B*,*C*,*D*,*S*, *qepA*, *oqx-A*,*B*, and *aac(6′)-Ib-cr*) (39, 40).

hvKP strains were tested for 15 different virulence-associated markers, including *fimH*, *mrkD*, *wabG*, *uge*, *wcaJ*, *magA*, *rmpA*, *rmpA2*, *entB*, *ybtS*, *iucA*, *iutA*, *iroN*, *allS*, and *kfuBC* by PCR (32, 39, 41). In this study, all PCRs were performed at least three times.

Significant differences in terms of AMR phenotypes, and carriage of resistance and virulence determinants between carbapenem-resistant and carbapenem-susceptible hvKPs were determined using χ^2^ or Fisher’s exact test.

### *In vitro* virulence assessment of the hvKP strains.

**(i) Biofilm-formation assay.** Biofilm-forming ability was assessed as described previously (42). Briefly, overnight cultures were adjusted to 0.5 McFarland, and 200 μL of bacterial suspensions were inoculated in 96-well plates. After incubation at 37°C for 24 h, biofilm was stained with 0.5% crystal violet, washed, and dissolved with 95% ethanol. Biofilm was quantified by measuring the absorbance at 595 nm (OD_595_). Strong biofilm was considered when the readings were at least three times greater than the negative control.

**(ii) Serum resistance assay.** Serum bactericidal activity was determined as described previously (43). Briefly, 25 μL of 10^6^ CFU/mL bacterial inoculum was added to 75 μL of pooled normal human serum (1:3 vol/vol ratio) donated by healthy volunteers. The mixture was incubated at 37°C, and viable counts were taken at 0, 1, 2, and 3 h. Heat-inactivated serum was also prepared by heating at 56°C for 30 min.

For *in vitro* virulence assessment, hvKP-K1 strain SB42 (*magA*^+^-*rmpA*^+^-*rmpA2*^+^-*iroN*^+^-*iucA*^+^-*iutA*^+^) was taken as the positive control, and K. pneumoniae ATCC 700603 as the negative control.

### Pulsed-field gel electrophoresis (PFGE) and multi-locus sequence typing (MLST).

PFGE was employed to determine clonal relatedness using the PulseNet protocol (http://www.cdc.gov/pulsenet/protocols.htm) in a CHEF-DR III apparatus (Bio-Rad Laboratories, Hercules and CA). DNA digestion was carried out for 12 h at 37°C using XbaI enzyme. Digested DNA fragments were electrophoresed for 20 h at 14°C at a 120° included angle, linear ramp factor with switch times of 2.2 and 54.2 s at 6 V/cm. Salmonella serotype Braenderup H9812 was used as the size determining marker. The PFGE results were interpreted according to Tenover criteria (44). The Dice coefficient (1% tolerance and 1% optimization) and the unweighted pair-group method with the arithmetic mean (UPGMA) were used for the cluster analysis, using the FPQuest Software v4.5 (Bio-Rad Laboratories).

For sequence typing analyses, sequences of 7 conserved housekeeping genes of K. pneumoniae (*gapA*, *infB*, *mdh*, *pgi*, *phoE*, *rpoB*, and *tonB*) were submitted to the multilocus sequence typing (MLST) database (https://bigsdb.pasteur.fr/cgi-bin/bigsdb/bigsdb.pl?db=pubmlst__klebsiella_seqdef).

### Bacterial conjugation.

Conjugation experiments were performed for CR-hvKP strains by a solid mating assay at 37°C, using the Escherichia coli J53 (azide-resistant) recipient (39). Transconjugants (Tc) were selected on LB agar plates supplemented with 1 mg/L ertapenem and 100 mg/L sodium azide (Sigma-Aldrich, St. Louis, USA). Putative transconjugants were screened for AMR determinants (including *bla*_NDM_), replicon types using PCR-based replicon typing (PBRT)-kit (Diatheva, Italy), and pLVPK-associated markers.

### DNA extraction, whole-genome sequencing, assembly, and annotation.

Based on the presence of pLVPK-associated markers (*rmpA*, *rmpA2*, *iroN*, *iucA*, and *iutA*), 2 NDM-1-producing CR-hvKPs, EN5180 (ST15-K54) and EN5289 (ST11-K2) strains were selected for WGS analysis. Genomic DNA (gDNA) of EN5180 and EN5289 were extracted using the QIAamp DNA minikit (Qiagen, Germany), according to the manufacturer’s instructions. The purity and concentration of the extracted gDNA were fluorometrically determined using Qubit 3.0 (Thermo Fisher Scientific). The genome sequencing library was prepared using NEBNext Ultra II DNA Library Prep Kit following the manufacturer’s instruction. Fragmented and adapter-tagged gDNA was amplified and sequenced on Illumina NextSeq 500 platform (Illumina Inc., San Diego, CA) with a read length of 151 bp. The sequence data was checked using FastQC v0.11.4, and adapters were trimmed using Cutadapt v2.5 (45, 46). The reads were *de novo* assembled using SPAdes v0.7.12-r1039 (47). Assessment of assembly metrics and genomic annotation were carried out using Quast v5.0.2 and Prokka v1.12 (48), respectively. Genomic islands were predicted using IslandViewer 4 (49).

### Analysis of the resistome, virulome, and other genetic elements.

Acquired AMR genes, plasmid replicons, and other elements in the studied genomes were determined using ResFinder (https://cge.cbs.dtu.dk/services/ResFinder/), PlasmidFinder (https://cge.food.dtu.dk/services/PlasmidFinder/), and the BIGSdb-Kp database (https://bigsdb.pasteur.fr/klebsiella/), respectively. Putative virulence factors were predicted using VRprofile (https://bioinfo-mml.sjtu.edu.cn/VRprofile/) with the BLASTp-based *Ha*-value > 0.64, which collected 2454 virulence determinants from the Virulence Factors Database (VFDB) (http://www.mgc.ac.cn/VFs/). Moreover, phage elements, CRISPR loci, and *in silico* K-antigen were identified using PHAST (http://phast.wishartlab.com/), CRISPRFinder (http://crispr.i2bc.paris-saclay.fr/Server/), and Kaptive (https://kaptive-web.erc.monash.edu/), respectively. The presence of pLVPK-like sequence in EN5180 and EN5289 was determined by progressiveMauve (http://darlinglab.org/mauve/user-guide/progressivemauve.html) using pLVPK (GenBank accession AY378100) as a reference.

### Comparative genomic and phylogenomic analysis.

For comparative genomics and phylogenomics, 11 publicly available reference genomes of hvKP [NTUH-2044 (GenBank accession NC_012731.1), 1084 (GenBank accession CP003785.1), ED23 (GenBank accession CP016814.1), ED2 (GenBank accession CP016813.1), RJF999 (GenBank accession CP014010.1), EN5275 (GenBank accession VINI00000000), RJF293 (GenBank accession CP014008.1), KCTC-2242 (GenBank accession CP002910.1), 1158 (GenBank accession CP006722.1), NUHL30457 (GenBank accession CP026586.1), and CG43 (GenBank accession CP006648.1)] were taken. Comparative BLAST+ and BLASTN analysis were carried out by Genome-to-Genome Distance Calculator (GGDC) v2.1 (http://ggdc.dsmz.de/) using the recommended formula 2 (identities/high-scoring pair lengths) and CGView server^BETA^ (http://cgview.ca/), respectively. For phylogenomic analysis, we generated alignments by mapping query genomes and reference genomes using bowtie2 on the REALPHY v1.12 server (https://realphy.unibas.ch/realphy/). The phylogenetic tree was constructed by the Interactive Tree Of Life (iTOL) server (https://itol.embl.de/) using the REALPHY-generated .phy file.

### *In silico* virulence plasmid analysis of EN5180 and EN5289.

From the draft genomes of EN5180 and EN5289, virulence plasmids were initially identified using MICRA pipeline (50). The annotation of the plasmids generated in MICRA were revalidated using RAST webserver (https://rast.nmpdr.org/). The mapping of plasmids were undertaken using Gview (https://server.gview.ca/), and pLVPK (GenBank accession AY378100) was used as a reference. Additionally, many of the protein-coding gene sequences present in plasmids were identified manually using blastx, and final maps were generated in Proksee server (https://proksee.ca/).

### Mouse lethality assay.

*In vivo* virulence of EN5180 and EN5289, carrying pLVPK-markers, was evaluated as described previously (51). Pathogen-free female 4 to 6-week-old BALB/c mice were infected with 100 μL of 1 × 10^5^ CFU/mL bacterial suspensions through the intraperitoneal route. Symptoms and mortality were monitored every 24 h up to 7 days. K. pneumoniae ATCC 700603 was used as a low virulence control, and K. pneumoniae strain SB42 was taken as the hypervirulent control. Survival curves were evaluated using Kaplan-Meier analysis and the log-rank test. *P < *0.05 was deemed significant. The animal experiments in this study were repeated three times.

### Data availability.

The raw sequencing reads of the CR-hvKP EN5180 and EN5289 strains were deposited in NCBI under BioProject number PRJNA684006. The genome sequences were submitted to the GenBank under the accession numbers JAELUV000000000 and JAELUW000000000 (Table 4).

## Supplementary Material

Reviewer comments

## References

[1] Viswanathan R, Singh AK, Basu S, Chatterjee S, Sardar S, Isaacs D. 2012. Multi-drug resistant gram negative bacilli causing early neonatal sepsis in India. Arch Dis Child Fetal Neonatal Ed 97:F182–F187. doi:10.1136/archdischild-2011-300097. 22155619

[2] Sands K, Carvalho MJ, Portal E, Thomson K, Dyer C, Akpulu C, Andrews R, Ferreira A, Gillespie D, Hender T, Hood K, Mathias J, Milton R, Nieto M, Taiyari K, Chan GJ, Bekele D, Solomon S, Basu S, Chattopadhyay P, Mukherjee S, Iregbu K, Modibbo F, Uwaezuoke S, Zahra R, Shirazi H, Muhammad A, Mazarati JB, Rucogoza A, Gaju L, Mehtar S, Bulabula ANH, Whitelaw A, Walsh TR, BARNARDS Group. 2021. Characterization of antimicrobial-resistant Gram-negative bacteria that cause neonatal sepsis in seven low- and middle-income countries. Nat Microbiol 6:512–523. doi:10.1038/s41564-021-00870-7. 33782558 PMC8007471

[3] Lee CR, Lee JH, Park KS, Kim YB, Jeong BC, Lee SH. 2016. Global dissemination of carbapenemase-producing *Klebsiella pneumoniae*: epidemiology, genetic context, treatment options, and detection methods. Front Microbiol 7:895. doi:10.3389/fmicb.2016.00895. 27379038 PMC4904035

[4] Wu W, Feng Y, Tang G, Qiao F, McNally A, Zong Z. 2019. NDM metallo-β-lactamases and their bacterial producers in health care settings. Clin Microbiol Rev 32:e00115-18. doi:10.1128/CMR.00115-18. 30700432 PMC6431124

[5] Navon-Venezia S, Kondratyeva K, Carattoli A. 2017. *Klebsiella pneumoniae*: a major worldwide source and shuttle for antibiotic resistance. FEMS Microbiol Rev 41:252–275. doi:10.1093/femsre/fux013. 28521338

[6] Escobar PJ, Olarte EN, Castro-Cardozo B, Valderrama MI, Garzón AM, Martinez de la Barrera L, Barrero BE, Marquez-Ortiz RA, Moncada GM, Vanegas GN. 2013. Outbreak of NDM-1-producing *Klebsiella pneumoniae* in a neonatal unit in Colombia. Antimicrob Agents Chemother 57:1957–1960. doi:10.1128/AAC.01447-12. 23357776 PMC3623329

[7] Zhang X, Li X, Wang M, Yue H, Li P, Liu Y, Cao W, Yao D, Liu L, Zhou X, Zheng R, Bo T. 2015. Outbreak of NDM-1-producing *Klebsiella pneumoniae* causing neonatal infection in a teaching hospital in mainland China. Antimicrob Agents Chemother 59:4349–4351. doi:10.1128/AAC.03868-14. 25941224 PMC4468712

[8] WHO. 2017. Global priority list of antibiotic-resistant bacteria to guide research, discovery, and development of new antibiotics. https://www.who.int/medicines/publications/global-priority-list-antibiotic-resistant-bacteria/en/.

[9] Centers for Disease Control and Prevention, National Center for Emerging Zoonotic and Infectious Diseases, National Center for HIV/AIDS, Viral Hepatitis, STD, and TB Prevention, National Center for Immunization and Respiratory Diseases. 2013. Antibiotic resistance threats in the United States. Centers for Disease Control and Prevention, Atlanta, GA. https://stacks.cdc.gov/view/cdc/20705.

[10] Shon AS, Bajwa RP, Russo TA. 2013. Hypervirulent (hypermucoviscous) *Klebsiella pneumoniae*: a new and dangerous breed. Virulence 4:107–118. doi:10.4161/viru.22718. 23302790 PMC3654609

[11] Paczosa MK, Mecsas J. 2016. *Klebsiella pneumoniae*: going on the offense with a strong defense. Microbiol Mol Biol Rev 80:629–661. doi:10.1128/MMBR.00078-15. 27307579 PMC4981674

[12] Russo TA, Marr CM. 2019. Hypervirulent *Klebsiella pneumoniae*. Clin Microbiol Rev 32:e00001-19. doi:10.1128/CMR.00001-19. 31092506 PMC6589860

[13] Walker KA, Miller VL. 2020. The intersection of capsule gene expression, hypermucoviscosity and hypervirulence in *Klebsiella pneumoniae*. Curr Opin Microbiol 54:95–102. doi:10.1016/j.mib.2020.01.006. 32062153 PMC8121214

[14] Choby JE, Howard-Anderson J, Weiss DS. 2020. Hypervirulent *Klebsiella pneumoniae* - clinical and molecular perspectives. J Intern Med 287:283–300. doi:10.1111/joim.13007. 31677303 PMC7057273

[15] Gu D, Dong N, Zheng Z, Lin D, Huang M, Wang L, Chan EW, Shu L, Yu J, Zhang R, Chen S. 2018. A fatal outbreak of ST11 carbapenem resistant hypervirulent *Klebsiella pneumoniae* in a Chinese hospital: a molecular epidemiological study. Lancet Infect Dis 18:37–46. doi:10.1016/S1473-3099(17)30489-9. 28864030

[16] Zhang Y, Jin L, Ouyang P, Wang Q, Wang R, Wang J, Gao H, Wang X, Wang H, Kang H, Gu B, Wang C, Cao B, Yang C, Jin L, Liao K, Zhang X, Ma X, Xie L, Zheng R, Zou H, Wang S, Pei F, Man S, Li W, Zhang Y, Cui Q, Jia X, Guo D, Fu Q, Zhang Z, Guo Z, Li Z, Xu Y, Ma X, Li Y, Jin Y, Liu Z, Zeng J, Li X, Zou C, Ji P, Jin C, Huang J, Tian J, Wu W, Xu X, Wen H, Yuan J, China Carbapenem-Resistant Enterobacteriaceae (CRE) Network. 2020. Evolution of hypervirulence in carbapenem resistant *Klebsiella pneumoniae* in China: a multicentre, molecular epidemiological analysis. J Antimicrob Chemother 75:327–336. doi:10.1093/jac/dkz446. 31713615

[17] Mukherjee S, Naha S, Bhadury P, Saha B, Dutta M, Dutta S, Basu S. 2020. Emergence of OXA-232-producing hypervirulent *Klebsiella pneumoniae* ST23 causing neonatal sepsis. J Antimicrob Chemother 75:2004–2006. doi:10.1093/jac/dkaa080. 32155265

[18] Harada S, Doi Y. 2018. Hypervirulent *Klebsiella pneumoniae*: a call for consensus definition and international collaboration. J Clin Microbiol 56:e00959-18. doi:10.1128/JCM.00959-18. 29950337 PMC6113475

[19] Mukherjee S, Mitra S, Dutta S, Basu S. 2021. Neonatal sepsis: the impact of carbapenem-resistant and hypervirulent *Klebsiella pneumoniae*. Front Med (Lausanne) 8:634349. doi:10.3389/fmed.2021.634349. 34179032 PMC8225938

[20] Li W, Sun G, Yu Y, Li N, Chen M, Jin R, Jiao Y, Wu H. 2014. Increasing occurrence of antimicrobial-resistant hypervirulent (hypermucoviscous) *Klebsiella pneumoniae* isolates in China. Clin Infect Dis 58:225–232. doi:10.1093/cid/cit675. 24099919

[21] Yu WL, Ko WC, Cheng KC, Lee HC, Ke DS, Lee CC, Fung CP, Chuang YC. 2006. Association between *rmpA* and *magA* genes and clinical syndromes caused by *Klebsiella pneumoniae* in Taiwan. Clin Infect Dis 42:1351–1358. doi:10.1086/503420. 16619144

[22] Jung SW, Chae HJ, Park YJ, Yu JK, Kim SY, Lee HK, Lee JH, Kahng JM, Lee SO, Lee MK, Lim JH, Lee CH, Chang SJ, Ahn JY, Lee JW, Park YG. 2013. Microbiological and clinical characteristics of bacteraemia caused by the hypermucoviscosity phenotype of *Klebsiella pneumoniae* in Korea. Epidemiol Infect 141:334–340. doi:10.1017/S0950268812000933. 22578630 PMC9152034

[23] Cubero M, Grau I, Tubau F, Pallarés R, Dominguez MA, Liñares J, Ardanuy C. 2016. Hypervirulent *Klebsiella pneumoniae* clones causing bacteraemia in adults in a teaching hospital in Barcelona, Spain (2007–2013). Clin Microbiol Infect 22:154–160. doi:10.1016/j.cmi.2015.09.025. 26454059

[24] Peirano G, Pitout JD, Laupland KB, Meatherall B, Gregson DB. 2013. Population-based surveillance for hypermucoviscosity *Klebsiella pneumoniae* causing community-acquired bacteremia in Calgary, Alberta. Can J Infect Dis Med Microbiol 24:e61–e64. doi:10.1155/2013/828741. 24421832 PMC3852459

[25] Guo Y, Wang S, Zhan L, Jin Y, Duan J, Hao Z, Lv J, Qi X, Chen L, Kreiswirth BN, Wang L, Yu F. 2017. Microbiological and Clinical Characteristics of Hypermucoviscous *Klebsiella pneumoniae* Isolates Associated with Invasive Infections in China. Front Cell Infect Microbiol 7:24. doi:10.3389/fcimb.2017.00024. 28203549 PMC5286779

[26] Zhao J, Chen J, Zhao M, Qiu X, Chen X, Zhang W, Sun R, Ogutu JO, Zhang F. 2016. Multilocus sequence types and virulence determinants of hypermucoviscosity-positive *Klebsiella pneumoniae* isolated from community-acquired infection cases in Harbin, North China. Jpn J Infect Dis 69:357–360. doi:10.7883/yoken.JJID.2015.321. 26743146

[27] Du P, Zhang Y, Chen C. 2018. Emergence of carbapenem-resistant hypervirulent *Klebsiella pneumoniae*. Lancet Infect Dis 18:23–24. doi:10.1016/S1473-3099(17)30625-4. 29102520

[28] Struve C, Roe CC, Stegger M, Stahlhut SG, Hansen DS, Engelthaler DM, Andersen PS, Driebe EM, Keim P, Krogfelt KA. 2015. Mapping the evolution of hypervirulent *Klebsiella pneumoniae*. mBio 6:e00630. doi:10.1128/mBio.00630-15. 26199326 PMC4513082

[29] Compain F, Vandenberghe A, Gominet M, Genel N, Lebeaux D, Ramahefasolo A, Podglajen I, Decré D. 2017. Primary osteomyelitis caused by an NDM-1-producing *K. pneumoniae* strain of the highly virulent sequence type 23. Emerg Microbes Infect 6:e57. doi:10.1038/emi.2017.43. 28634354 PMC5520317

[30] Liu BT, Su WQ. 2019. Whole genome sequencing of NDM-1-producing serotype K1 ST23 hypervirulent *Klebsiella pneumoniae* in China. J Med Microbiol 68:866–873. doi:10.1099/jmm.0.000996. 31107201

[31] Ye M, Tu J, Jiang J, Bi Y, You W, Zhang Y, Ren J, Zhu T, Cao Z, Yu Z, Shao C, Shen Z, Ding B, Yuan J, Zhao X, Guo Q, Xu X, Huang J, Wang M. 2016. Clinical and genomic analysis of liver abscess-causing *Klebsiella pneumoniae* identifies new liver abscess-associated virulence genes. Front Cell Infect Microbiol 6:165. doi:10.3389/fcimb.2016.00165. 27965935 PMC5126061

[32] Yao B, Xiao X, Wang F, Zhou L, Zhang X, Zhang J. 2015. Clinical and molecular characteristics of multi-clone carbapenem-resistant hypervirulent (hypermucoviscous) *Klebsiella pneumoniae* isolates in a tertiary hospital in Beijing, China. Int J Infect Dis 37:107–112. doi:10.1016/j.ijid.2015.06.023. 26141415

[33] Yang X, Dong N, Chan EW, Zhang R, Chen S. 2021. Carbapenem resistance-encoding and virulence-encoding conjugative plasmids in *Klebsiella pneumoniae*. Trends Microbiol 29:65–83. doi:10.1016/j.tim.2020.04.012. 32448764

[34] Diago-Navarro E, Calatayud-Baselga I, Sun D, Khairallah C, Mann I, Ulacia-Hernando A, Sheridan B, Shi M, Fries BC. 2017. Antibody-based immunotherapy to treat and prevent infection with hypervirulent *Klebsiella pneumoniae*. Clin Vaccine Immunol 24:e00456-16. doi:10.1128/CVI.00456-16. 27795303 PMC5216427

[35] Clinical and Laboratory Standards Institute. 2020. Performance standards for antimicrobial susceptibility testing: thirtieth informational supplement M100-S30. CLSI, Wayne, PA.

[36] Fang CT, Lai SY, Yi WC, Hsueh PR, Liu KL, Chang SC. 2007. *Klebsiella pneumoniae* genotype K1: an emerging pathogen that causes septic ocular or central nervous system complications from pyogenic liver abscess. Clin Infect Dis 45:284–293. doi:10.1086/519262. 17599305

[37] Turton JF, Perry C, Elgohari S, Hampton CV. 2010. PCR characterization and typing of *Klebsiella pneumoniae* using capsular type-specific, variable number tandem repeat and virulence gene targets. J Med Microbiol 59:541–547. doi:10.1099/jmm.0.015198-0. 20110386

[38] Wiegand I, Hilpert K, Hancock REW. 2008. Agar and broth dilution methods to determine the minimal inhibitory concentration (MIC) of antimicrobial substances. Nat Protoc 3:163–175. doi:10.1038/nprot.2007.521. 18274517

[39] Mukherjee S, Bhattacharjee A, Naha S, Majumdar T, Debbarma SK, Kaur H, Dutta S, Basu S. 2019. Molecular characterization of NDM-1-producing *Klebsiella pneumoniae* ST29, ST347, ST1224, and ST2558 causing sepsis in neonates in a tertiary care hospital of North-East India. Infect Genet Evol 69:166–175. doi:10.1016/j.meegid.2019.01.024. 30677535

[40] Naha S, Sands K, Mukherjee S, Roy C, Rameez MJ, Saha B, Dutta S, Walsh TR, Basu S. 2020. KPC-2-producing *Klebsiella pneumoniae* ST147 in a neonatal unit: clonal isolates with differences in colistin susceptibility attributed to AcrAB-TolC pump. Int J Antimicrob Agents 55:105903. doi:10.1016/j.ijantimicag.2020.105903. 31954832

[41] Fu L, Huang M, Zhang X, Yang X, Liu Y, Zhang L, Zhang Z, Wang G, Zhou Y. 2018. Frequency of virulence factors in high biofilm formation *bla*_KPC-2_ producing *Klebsiella pneumoniae* strains from hospitals. Microb Pathog 116:168–172. doi:10.1016/j.micpath.2018.01.030. 29360567

[42] Wu MC, Lin TL, Hsieh PF, Yang HC, Wang JT. 2011. Isolation of genes involved in biofilm formation of a *Klebsiella pneumoniae* strain causing pyogenic liver abscess. PLoS One 6:e23500. doi:10.1371/journal.pone.0023500. 21858144 PMC3155550

[43] Podschun R, Sievers D, Fischer A, Ullmann U. 1993. Serotypes, hemagglutinins, siderophore synthesis, and serum resistance of *Klebsiella* isolates causing human urinary tract infections. J Infect Dis 168:1415–1421. doi:10.1093/infdis/168.6.1415. 7902383

[44] Tenover FC, Arbeit RD, Goering RV, Mickelsen PA, Murray BE, Persing DH, Swaminathan B. 1995. Interpreting chromosomal DNA restriction patterns produced by pulsed-field gel electrophoresis: criteria for bacterial strain typing. J Clin Microbiol 33:2233–2239. doi:10.1128/jcm.33.9.2233-2239.1995. 7494007 PMC228385

[45] Andrews S. 2010. FastQC: a quality control tool for high throughput sequence data. http://www.bioinformatics.babraham.ac.uk/projects/fastqc.

[46] Martin M. 2011. Cutadapt removes adapter sequences from high-throughput sequencing reads. EMBnet j 17:10–12. doi:10.14806/ej.17.1.200.

[47] Bankevich A, Nurk S, Antipov D, Gurevich AA, Dvorkin M, Kulikov AS, Lesin VM, Nikolenko SI, Pham S, Prjibelski AD, Pyshkin AV, Sirotkin AV, Vyahhi N, Tesler G, Alekseyev MA, Pevzner PA. 2012. SPAdes: a new genome assembly algorithm and its applications to single-cell sequencing. J Comput Biol 19:455–477. doi:10.1089/cmb.2012.0021. 22506599 PMC3342519

[48] Seemann T. 2014. Prokka: rapid prokaryotic genome annotation. Bioinformatics 30:2068–2069. doi:10.1093/bioinformatics/btu153. 24642063

[49] Bertelli C, Laird MR, Williams KP, Lau BY, Hoad G, Winsor GL, Brinkman FSL, Simon Fraser University Research Computing Group. 2017. IslandViewer 4: expanded prediction of genomic islands for larger-scale datasets. Nucleic Acids Res 45:W30–W35. doi:10.1093/nar/gkx343. 28472413 PMC5570257

[50] Caboche S, Even G, Loywick A, Audebert C, Hot D. 2017. MICRA: an automatic pipeline for fast characterization of microbial genomes from high-throughput sequencing data. Genome Biol 18:233. doi:10.1186/s13059-017-1367-z. 29258574 PMC5738152

[51] Chen Y, Marimuthu K, Teo J, Venkatachalam I, Cherng BPZ, De Wang L, Prakki SRS, Xu W, Tan YH, Nguyen LC, Koh TH, Ng OT, Gan YH. 2020. Acquisition of plasmid with carbapenem-resistance gene *bla*_KPC2_ in hypervirulent *Klebsiella pneumoniae*, Singapore. Emerg Infect Dis 26:549–559. doi:10.3201/eid2603.191230. 32091354 PMC7045839

