# Correction for Mukherjee et al., “Hypervirulent *Klebsiella pneumoniae* Causing Neonatal Bloodstream Infections: Emergence of NDM-1-Producing Hypervirulent ST11-K25 and ST15-K54 Strains Possessing pLVPK-Associated Markers”

**DOI:** 10.1128/spectrum.00346-25

**Published:** 2025-03-17

**Authors:** Subhankar Mukherjee, Punyasloke Bhadury, Shravani Mitra, Sharmi Naha, Bijan Saha, Shanta Dutta, Sulagna Basu

## AUTHOR CORRECTION

Volume 11, no. 2, e04121-22, 2023, https://doi.org/10.1128/spectrum.04121-22. The title should read as given in this correction, and “ST11-K2” should read “ST11-K25” throughout. Although based on short-read sequencing strain EN5289 was identified as belonging to the ST11-K2 serotype, long-read sequencing indicates that it belongs to the ST11-K25 serotype. The results and conclusions presented in the original paper are valid if the serotype is replaced throughout the text.

