# Supplementary material for: Hypervirulent Klebsiella pneumoniae Causing Neonatal Bloodstream Infections: Emergence of NDM-1-Producing Hypervirulent ST11-K2 and ST15-K54 Strains Possessing pLVPK-Associated Markers
Source: Microbiol Spectr. 2023 Feb 8;11(2):e04121-22. doi: 10.1128/spectrum.04121-22 (PMC10101084; doi:10.1128/spectrum.04121-22)
Supplement: Supplemental file 1 — Supplemental material. Download spectrum.04121-22-s0001.pdf, PDF file, 1.3 MB [file spectrum.04121-22-s0001.pdf]

Supplementary material

Hypervirulent *Klebsiella pneumoniae* causing neonatal bloodstream infections: emergence of NDM-1-producing hypervirulent ST11-K2 and ST15-K54 strains possessing pLVPK-associated markers

Subhankar Mukherjee[a*], Punyasloke Bhadury[b], Shravani Mitra[a], Sharmi Naha[a], Bijan Saha[c], Shanta Dutta[a], Sulagna Basu[a#]

Affiliations:

[a] *Division of Bacteriology, ICMR-National Institute of Cholera and Enteric Diseases. Kolkata, West Bengal, India.*

[b] *Integrative Taxonomy and Microbial Ecology Research Group, Department of Biological Sciences, Indian Institute of Science Education and Research Kolkata, Mohanpur, Nadia, West Bengal, India.*

[c] *Department of Neonatology, Institute of Postgraduate Medical Education & Research, SSKM Hospital, Kolkata, West Bengal, India.*

* ***Present address****: Department of Zoology, Government General Degree College, Singur, Hooghly, West Bengal, India.*

Keywords: *Klebsiella pneumoniae*, neonatal sepsis, antibiotic resistance, carbapenem resistance, hypervirulence, India.

# Corresponding author:

Sulagna Basu, Division of Bacteriology, ICMR-National Institute of Cholera and Enteric Diseases, P-33, C.I.T. Road, Scheme XM, Beliaghata, Kolkata-700 010, West Bengal, India.

E-mail address: supabasu@yahoo.co.in; basus.niced@gov.in

Telephone: +91-33-2353 7469/7470, 23705533/4478/0448; ext: 3055. Fax: +91-33-2363, 2370 5066

Supplementary Figure legends

Supplementary Figure 1. Antibiotic susceptibility profile of neonatal septicemic *K. pneumoniae* (n = 107) against different antimicrobials. More than 50% of strains were resistant to nine different antimicrobials.

Supplementary Figure 2. progressiveMauve alignment between the publicly available sequence of pLVPK (GenBank accession AY378100) with CR-hvKP strains EN5180 (GenBank accession JAELUV000000000) **(a)** and EN5289 (GenBank accession JAELUW000000000) **(b)** confirmed the presence of virulence plasmid-like sequence in the studied genomes.

Supplementary Figure 3. Analysis of prophage elements in the CR-hvKP EN5180 and EN5289 genomes. PHAST algorithm identified six (2 were intact and 4 were incomplete) and seventeen (4 were intact, 10 were incomplete, and 3 were questionable) prophage regions in the EN5180 and EN5289, respectively. Sizes of the prophage elements varied from 8.2 to 120.1 kb (for EN5180) and 6.5 to 62.6 kb (for EN5289). Structural elements of the identified prophages recovered from EN5180 **(a)** and EN5289 **(b)** are represented.

Supplementary Figure 4. Analysis of CRISPR arrays in the CR-hvKP EN5180 and EN5289 genomes. CRISPRFinder revealed the presence of three intact CRISPRs (two CRISPR 1 arrays and one CRISPR 2) and one complete CRISPR array (CRISPR 1) in the genome of EN5180 and EN5289, respectively. The identified CRISPRs length, numbers and sequences of direct repeats and spacers elements are represented in **4a** (for EN5180) and **4b** (for EN5289).

Supplementary Figure 5. Whole genome-based phylogenetic analysis (via REALPHY v1.12 and iTOL) of CR-hvKP EN5180 and EN5289 with 11 other reported hvKP genomes from China, Taiwan, Korea, and India. The phylogenetic tree demonstrated that the genome of CR-hvKP EN5180 and EN5289 are similar to that of the other reported hvKP strains. The table portrays the microbiological features of the hvKP genomes. Lighter red-colored shades indicate our sequenced genomes of interest.

Supplementary Table 1. Assessment of *in vitro* virulence of the studied hvKP strains (n = 28)

Strains	Capsular type	ST	Virulence determinants	Biofilm forming capability (OD_{595})	Susceptibility to human serum
**Carbapenem-resistant hvKP (n = 9)**					
EN5180	K54	15	*fimH*, *mrkD*, *wabG*, *uge*, *wcaJ*, *rmpA*, *rmpA2*, *entB*, *ybtS*, *iucA*, *iutA*, *iroN*, *allS*, *kfuBC*	Strong (1.01)	Resistant
EN5187	K54	307	*fimH*, *mrkD*, *wabG*, *uge*, *wcaJ*, *entB*, *ybtS*, *kfuBC*	Strong (0.99)	Resistant
EN5199	K2	14	*fimH*, *mrkD*, *wabG*, *uge*, *wcaJ*, *entB*, *ybtS*, *kfuBC*	Moderate (0.69)	Resistant
EN5206	K2	14	*fimH*, *mrkD*, *wabG*, *uge*, *wcaJ*, *entB*, *ybtS*, *kfuBC*	Moderate (0.72)	Resistant
EN5289	K2	11	*fimH*, *mrkD*, *wabG*, *uge*, *wcaJ*, *rmpA*, *rmpA2*, *entB*, *ybtS*, *iucA*, *iutA*, *iroN*, *allS*, *kfuBC*	Strong (1.23)	Resistant
EN5298	K2	15	*fimH*, *mrkD*, *wabG*, *uge*, *wcaJ*, *entB*, *ybtS*, *kfuBC*	Strong (0.89)	Resistant
EN5327	K2	65	*fimH*, *mrkD*, *wabG*, *uge*, *wcaJ*, *entB*, *ybtS*, *allS*, *kfuBC*	Strong (1.07)	Resistant
EN5329	K2	65	*fimH*, *mrkD*, *wabG*, *uge*, *wcaJ*, *entB*, *ybtS*, *allS*, *kfuBC*	Strong (1.04)	Resistant
EN5337	K20	268	*fimH*, *mrkD*, *wabG*, *uge*, *wcaJ*, *entB*, *ybtS*, *kfuBC*	Low (0.39)	Susceptible
**Carbapenem-susceptible hvKP (n = 19)**					
EN5190	K54	29	*fimH*, *mrkD*, *kfuBC*	Low (0.30)	Resistant
EN5198	K2	520	*fimH*, *mrkD*, *wabG*, *entB*, *kfuBC*	Strong (0.87)	Resistant
EN5211	K2	14	*fimH*, *mrkD*, *wabG*, *uge*, *entB*, *kfuBC*	Moderate (0.61)	Resistant
EN5221	K2	14	*mrkD*, *wabG*, *uge*, *entB*, *kfuBC*	Moderate (0.54)	Resistant
EN5230	K2	14	*wabG*, *uge*, *entB*, *kfuBC*	Low (0.35)	Susceptible
EN5233	K2	520	*fimH*, *mrkD*, *wabG*, *uge*, *entB*, *kfuBC*	Strong (0.95)	Resistant
EN5235	K2	520	*fimH*, *mrkD*, *wabG*, *uge*, *entB*, *kfuBC*	Strong (0.97)	Resistant
EN5236	K2	520	*fimH*, *mrkD*, *wabG*, *uge*, *entB*, *kfuBC*	Strong (0.89)	Resistant
EN5245	K54	1308	-	Low (0.20)	Susceptible
EN5259	K2	29	*fimH*, *mrkD*, *wabG*, *uge*, *entB*, *kfuBC*	Low (0.43)	Resistant
EN5272	K2	14	*fimH*, *mrkD*, *wabG*, *uge*, *entB*, *kfuBC*	Moderate (0.67)	Resistant
EN5291	K2	14	*fimH*, *mrkD*, *wabG*, *uge*, *entB*, *kfuBC*	Moderate (0.55)	Resistant
EN5292	K2	14	*fimH*, *mrkD*, *wabG*, *uge*, *entB*, *kfuBC*	Low (0.41)	Resistant
EN5303	K57	1035	*wabG*, *entB*, *allS*, *kfuBC*	Low (0.36)	Resistant
EN5306	K57	1035	*wabG*, *allS*, *kfuBC*	Low (0.29)	Resistant

EN5310	K54	711	*fimH*, *mrkD*, *wabG*, *uge*, *ybtS*	Strong (0.82)	Susceptible
EN5315	K2	14	*fimH*, *mrkD*, *wabG*, *uge*, *entB*, *kfuBC*	Moderate (0.65)	Susceptible
EN5323	K57	1035	*wabG*, *allS*, *kfuBC*	Low (0.31)	Resistant
EN5334	K57	2343	*fimH*, *mrkD*, *wabG*, *uge*, *ybtS*, *allS*	Strong (0.88)	Susceptible

Strong biofilm was considered when OD_S > $3OD_N$ and moderate biofilm was considered when OD_S > $2OD_N$. OD_S – Optical Density of the sample and OD_N – Optical Density (OD_{595} = 0.27) of the negative control (*K. pneumoniae* ATCC 700603). Optical Density of the hvKP-K1 positive control strain SB42 (OD_{595} = 1.36)

Supplementary Table 2. Tabular representation of prophage elements detected in CR-hvKP EN5180 (a) and EN5289 (b)

Region	Region_Length	Completeness	Score	CDS	Region_Position	Possible phage	GC_Percentage
1	8.2Kb	incomplete	20	11	670746-678950	PHAGE_Erwini_vB_EamM_Kwan_NC_031010, ...	50.31%
2	23.9Kb	intact	150	26	684305-708209	PHAGE_Entero_P88_NC_026014, ...	53.97%
3	120.1Kb	intact	150	117	3247004-3367110	PHAGE_Salmon_SSU5_NC_018843, ...	49.03%
4	8.2Kb	incomplete	30	10	4966121-4974324	PHAGE_Salmon_SJ46_NC_031129, ...	49.90%
5	8.5Kb	incomplete	30	15	5224489-5233015	PHAGE_Citrob_vB_CfrM_CfP1_NC_031057, ...	47.84%
6	8.5Kb	incomplete	50	11	5974908-5983461	PHAGE_Salmon_SJ46_NC_031129, ...	51.20%

(a)

Region	Region_Length	Completeness	Score	CDS	Region_Position	Possible phage	GC_Percentage
1	40.1Kb	questionable	80	25	2246918-2287108	PHAGE_Salmon_ST64B_NC_004313, ...	53.96%
2	21.3Kb	incomplete	40	9	3178832-3200160	PHAGE_Iodoba_phiPLPE_NC_011142, ...	53.15%
3	25.6Kb	incomplete	30	24	3350313-3375987	PHAGE_Entero_Tyrion_NC_031077, ...	54.19%
4	21.1Kb	incomplete	20	32	3482303-3503458	PHAGE_Entero_UAB_Phi20_NC_031019, ...	53.86%
5	21.8Kb	incomplete	30	10	3713823-3735714	PHAGE_Entero_P4_NC_001609, ...	55.08%
6	32.5Kb	questionable	80	45	4024674-4057265	PHAGE_Salmon_103203_sal5_NC_031946, ...	51.22%
7	37.2Kb	incomplete	60	29	4320310-4357534	PHAGE_Escher_phiV10_NC_007804, ...	54.86%
8	62.6Kb	intact	150	62	4937703-5000312	PHAGE_Salmon_RE_2010_NC_019488, ...	52.93%
9	8.2Kb	incomplete	30	9	5148802-5157005	PHAGE_Salmon_SJ46_NC_031129, ...	49.91%
10	9.2Kb	incomplete	50	16	5256497-5265791	PHAGE_Gordon_Schwabeltier_N	53.82%

						C_031255, ...	
11	12.5Kb	questionable	75	18	5284565-5297068	PHAGE_Entero_P4_NC_001609, ...	50.25%
12	6.5Kb	incomplete	30	10	5385012-5391512	PHAGE_Burkho_KL3_NC_015266, ...	47.64%
13	26Kb	intact	140	38	5552252-5578266	PHAGE_Salmon_Fels_2_NC_010463, ...	50.18%
14	16.4Kb	incomplete	60	24	5819737-5836158	PHAGE_Salmon_SEN34_NC_028699, ...	52.74%
15	11.5Kb	incomplete	40	19	5856945-5868495	PHAGE_Salmon_SJ46_NC_031129, ...	51.53%
16	45.1Kb	intact	150	55	5893197-5938333	PHAGE_Salmon_SEN34_NC_028699, ...	51.76%
17	30.7Kb	intact	100	18	5935119-5965903	PHAGE_Salmon_SJ46_NC_031129, ...	51.34%

(b)

Red, green, and grey colors indicate the presence of intact, questionable, and incomplete phages, respectively.

Supplementary Table 3. BLAST+ comparison of hypervirulent *K. pneumoniae* genomes using Genome-to-Genome Distance Calculator.

Query genome	Reference genomes	GenBank accession numbers of reference genomes	Genome-to-Genome Distance Calculator (GGDC) output<br>Formula 2 (identities/high-scoring pair lengths)				G+C difference
			dDDH* values (%)	Model C.I.†	Distance	Prob. dDDH>= 70%	
K. pneumoniae EN5180	*K. pneumoniae* 1084	CP003785.1	93.9	[92.1 - 95.3%]	0.0079	96.94	1.21
K. pneumoniae EN5180	*K. pneumoniae* ED2	CP016813.1	94.1	[92.3 - 95.4%]	0.0077	96.98	1.03
K. pneumoniae EN5180	*K. pneumoniae* ED23	CP016814.1	94	[92.2 - 95.4%]	0.0078	96.96	1.41
K. pneumoniae EN5180	*K. pneumoniae* NTUH-2044	NC_012731.1	93.9	[92.1 - 95.3%]	0.0079	96.93	1.22
K. pneumoniae EN5180	*K. pneumoniae* RJF999	CP014010.1	94	[92.2 - 95.4%]	0.0078	96.96	1.37
K. pneumoniae EN5180	*K. pneumoniae* EN5275	VINI00000000	93.3	[91.5 - 94.8%]	0.0085	96.79	0.49
K. pneumoniae	*K. pneumoniae*	CP026586.1	93.9	[92.1 - 95.3%]	0.0078	96.94	1.35

EN5180	NUHL30457						
K. pneumoniae EN5180	*K. pneumoniae* CG43	CP006648.1	94.2	[92.5 - 95.6%]	0.0075	97.02	1.48
K. pneumoniae EN5180	*K. pneumoniae* KCTC-2242	CP002910.1	93.8	[92 - 95.2%]	0.008	96.91	1.33
K. pneumoniae EN5180	*K. pneumoniae* 1158	CP006722.1	93.3	[91.4 - 94.8%]	0.0086	96.77	1.3
K. pneumoniae EN5180	*K. pneumoniae* RJF293	CP014008.1	94.3	[92.6 - 95.6%]	0.0074	97.03	1.24
K. pneumoniae EN5289	*K. pneumoniae* 1084	CP003785.1	93.3	[91.4 - 94.8%]	0.0086	96.77	0.76
K. pneumoniae EN5289	*K. pneumoniae* ED2	CP016813.1	93.4	[91.5 - 94.8%]	0.0085	96.8	0.57
K. pneumoniae EN5289	*K. pneumoniae* ED23	CP016814.1	93.3	[91.4 - 94.8%]	0.0085	96.79	0.96
K. pneumoniae EN5289	*K. pneumoniae* NTUH-2044	NC_012731.1	93.5	[91.6 - 94.9%]	0.0083	96.82	0.77
K. pneumoniae EN5289	*K. pneumoniae* RJF999	CP014010.1	92.9	[91 - 94.5%]	0.0089	96.68	0.92
K. pneumoniae EN5289	*K. pneumoniae* EN5275	VINI00000000	93.2	[91.3 - 94.7%]	0.0086	96.77	0.03
K. pneumoniae EN5289	*K. pneumoniae* NUHL30457	CP026586.1	93.8	[91.9 - 95.2%]	0.008	96.9	0.9
K. pneumoniae EN5289	*K. pneumoniae* CG43	CP006648.1	94.1	[92.3 - 95.5%]	0.0076	96.99	1.02
K. pneumoniae EN5289	*K. pneumoniae* KCTC-2242	CP002910.1	93.4	[91.6 - 94.9%]	0.0084	96.82	0.88
K. pneumoniae EN5289	*K. pneumoniae* 1158	CP006722.1	93.4	[91.5 - 94.9%]	0.0084	96.81	0.84
K. pneumoniae EN5289	*K. pneumoniae* RJF293	CP014008.1	93.7	[91.8 - 95.1%]	0.0081	96.87	0.78

*dDDH, Digital DNA-DNA hybridization; †C.I., confidence intervals.

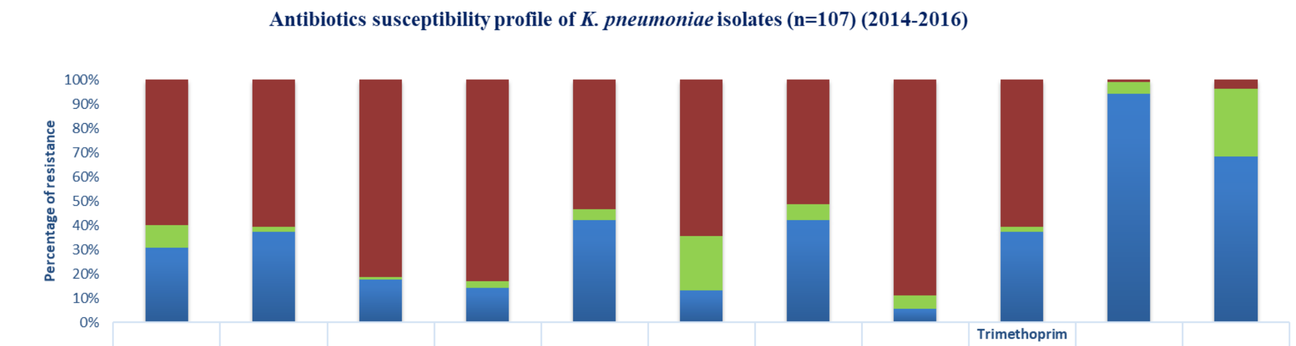

	Amikacin	Gentamicin	Aztreonam	Cefotaxime	Cefoxitin	Ciprofloxacin	Meropenem	Piperacillin	Trimethoprim/sulfamethoxazole	Tigecycline	Colistin
Resistance	64	65	87	89	57	69	55	95	65	1	4
Intermediate	10	2	1	3	5	24	7	6	2	5	30
Susceptible	33	40	19	15	45	14	45	6	40	101	73

Supplementary Figure 1

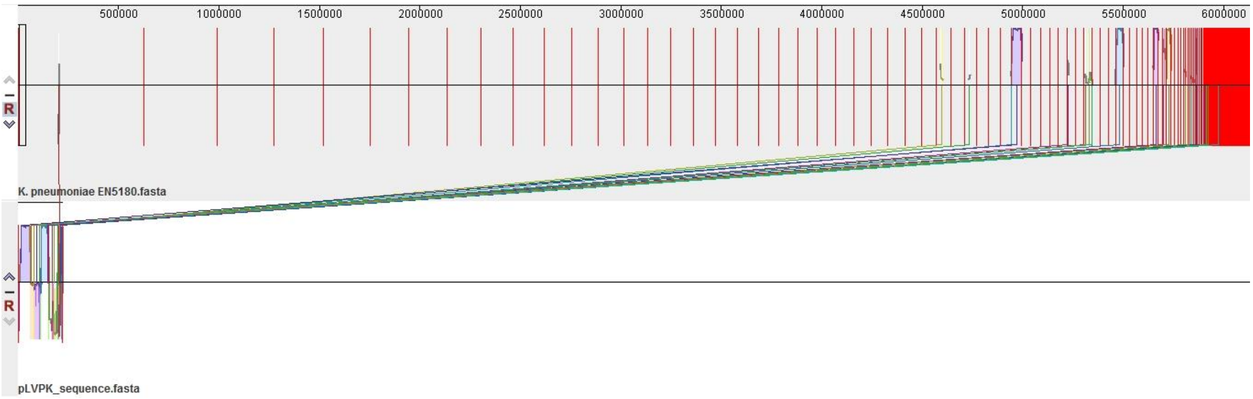

(a)

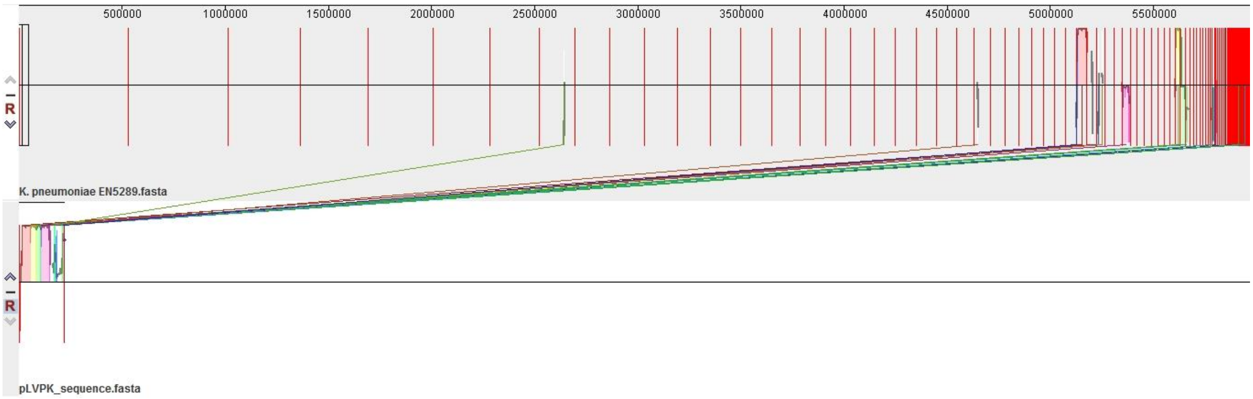

(b)

Supplementary Figure 2

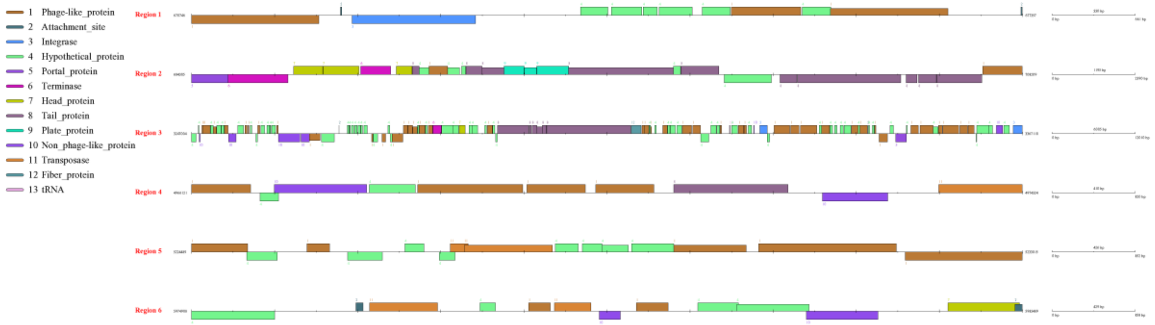

(a)

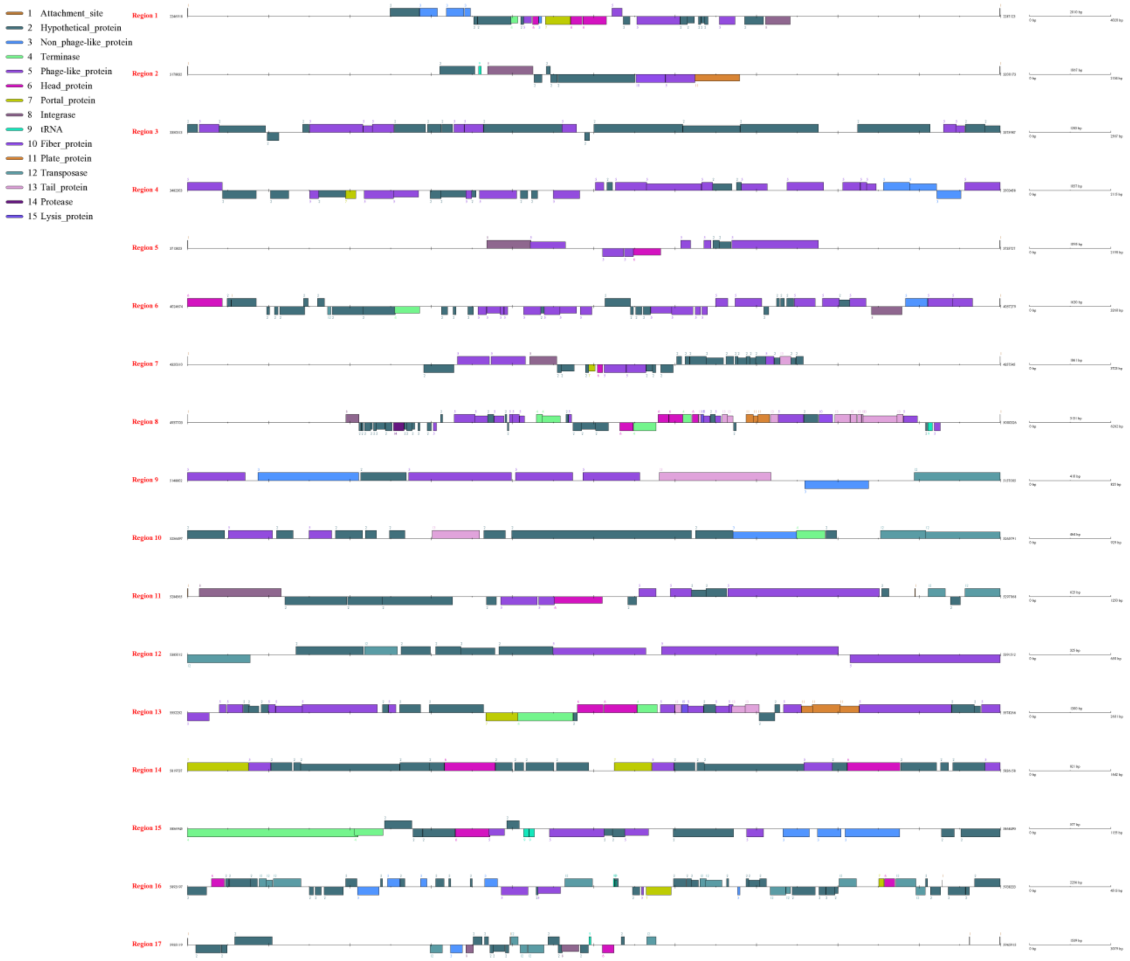

(b)

Supplementary Figure 3

CRISPR id : tmp_9_Crispr_1

- CRISPR start position : 14097 ---------- CRISPR end position : 14804 ---------- CRISPR length : 707
- DR consensus : AGAAACACCCCCACGTGCGTGGGGAAGAC
- DR length : 29 Number of spacers : 11

14097	AAGAAACACCCCACGTACATGGTGAAGAC	AGAACGAATGCCAGCGCTGGTACGGCGCGTCGTGGATTCC	14165
14166	AGAAACACCCCCACGTGCGTGGGGAAGAC	TGCCGGATATCATCACCGCGATTAAACGGCGG	14226
14227	AGAAACACCCCCACGTGCGTGGGGAAGAC	CATTCATAACAAAACCGCTTTTACTAGATGAG	14287
14288	GGAAACACCCCCACGTGCGTGGGGAAGAC	GCGGTGAACCTTGGGGCGTCTCCTCGCGAACT	14348
14349	AGAAACACCCCCACGTGCGTGGGGAAGAC	GCTAACCAGTGGATAGAGCACTATGTGACGAC	14409
14410	AGAAACACCCCCACGTGCGTGGGGAAGAC	GCTACTGCATCCACGGCGTACATGCTCAGTGT	14470
14471	AGAAACATCCCCACGTGCGTGGGGAAGAC	TGCACCCCTTCACGCCAAATGCAGCCGTATCT	14531
14532	AGAAACACCCCCACGTGCGTGGGGAAGAC	GCAGTCGATATCGTCTCGACAGTGACAGTGCC	14592
14593	AGAAACACCCCCACGTGCGTGGGGAAGAC	TACCTGTCAGTGAAAAAACTGTCCCGCCAACG	14653
14654	GGAAACACCCCCACGTGCGTGGGGAAGAC	TGCCGCAGACCGGGCAGCGCGGTATGTCCGTC	14714
14715	AGAAACACCCCCACGTGCGTGGGGAAGAC	GCCACCGGCGGCGCCGAGATCGGGCCATGCGA	14775
14776	AGAAACACCCCCACGTGCGTGGGGAAGAC		14804

CRISPR id : tmp_9_Crispr_2

- CRISPR start position : 23557 ---------- CRISPR end position : 23952 ---------- CRISPR length : 395
- DR consensus : AGAAACACCCCCACGCGTGTGGGGAAGAC
- DR length : 29 Number of spacers : 6

23557	GAAAACACCTCCACGCATGTGGGGAAGAC	AGGATAGAGCCAAATCCGCTCACACGTGATGA	23617
23618	AGAAACACCCCCACACATGTGGGGAAGAC	CCGGCATCCGTCAGCTCGACGGCCAGCTGCAG	23678
23679	GGAAACACCCCCACGCGTGTGGGGGAAGA	CACGTGATCGCCCTGGCGCGGACGCCGGGAGGT	23740
23741	AGAAACACCCCCACGCGTGTGGGGAAGAC	ATGGTGCGACTGTAGAATCCTCACCATGCACG	23801
23802	AGAAACACCCCCACGCGTGTGGGGAAGAC	GATAATCCCGTCAGGTTGTGACTCTGCACGAT	23862
23863	AGAAACACCCCCACGCGTGTGGGGAAGAC	CCGGTTAGCAGGCCGGTGATTGCATTGGAGGC	23923
23924	AGAAACACCCCCACGCGTGTGGGGAAGAC		23952

CRISPR id : tmp_69_Crispr_1

- CRISPR start position : 1497 ---------- CRISPR end position : 2739 ---------- CRISPR length : 1242
- DR consensus : GTATTCCCCCCGCATGCGGGGGTTATCGG
- DR length : 29 Number of spacers : 20

1497	GTATTCCCCCCGTGTGCGGGGGTTATCGG	TGGTGCTCTCAACCGTCACCCGCTGGCTGGAA	1557
1558	GTATTCCCCCCGTGTGCGGGGGTTATCGG	TCGTGTTGTCCACGGTTACCCGCTGGCTGGAA	1618
1619	GTATTCCCCCCGTGTGCGGGGGTTATTGG	TTACCAATGGGGAAAAATCTTCATTTGTAAAT	1679
1680	GTATTCCCCCCGCATGCGGGGGTTATCGG	AACATCAGTGGAAATCCACTGCGGC	1733
1734	GTATTCTCCCCGCATGCGGGGGTTATCGG	CGAAAACGGCAACCTTCATAAAAACGTCTTTT	1794
1795	GTATTCCCCCCGTGTGCGGGGGTTATCGG	CCGAGATTGAGTAAAGCAAAGTAACGGCGGTG	1855
1856	GTATTCCCCCCGCATGCGGGGGTTATCGG	TGTGTGTTGGCGTTCGTTAAATATTGTTAGTA	1916
1917	GTATTCCCCCCGCATGCGGGGGTTATCGG	CGGTAACGCAAATGTGATCCGATGTCGTCAGG	1977
1978	GTATTCCCCTCGCTTACGGGGGTTATCGG	AACAATTTGAAGTTTCTGCGCCAGGTCGTTTC	2038
2039	GTATTCCCCCCGCATGCGGGGGTTATCGG	CAGGTTATACTGGCAAAACGTCGATGGCTCT	2098

2099	**CGTATTCCCCCGCTTGCGGGGGTTATCGG**	TAAATCAGCAAATATTGTTGTCTACCGTGTCG	2159
2160	**GTATTCCCCCCGTCTGCGGGGGTTATCGG**	AGCAGTTCGAGGAATAGTGACAGGCAGTGCAG	2220
2221	**GTATTCCCCCCGTGTGCGGGGGTTATCGG**	TTAATGTTTTGTTAATTTATGAGTGTGGTGATG	2282
2283	**TATTCCCCCCCGACTGCGGGGGTTATCGG**	AGCAAATCGAAAATCCGGCTGTTTGAAAAATGGT	2345
2346	**ATTCCCCCCCCGCTTACGGGGGTTATCGC**	CATGAGCCTGCGCACTCTGACGCGCACCTGCT	2406
2407	**GTATTCCCCCCGCATGCGGGGGTTATCGG**	TGAGGCTGCTGACGGAGAATTGGACCTGTTC	2467
2468	**GTATTCCCCCCGCACGCGGGGGTTATCGG**	CCGACCCGGTGCCCAGGAGAACTGGCTGAATA	2528
2529	**GTATTCCCCCCGCGTACGGGGGTTATCGG**	TTCCCTGCACTAAGACGCTGGTGGTCGCCAC	2588
2589	**GTACTCCCCCCGCATGCGGGGGTTATTGG**	CCGGTTCGGATTTTGCGAAACAGGTGCAGGGC	2649
2650	**GTATTCCCCCCGCTTGCGGGGGTTATCGG**	GGCATGAGCGAGAACCACTGCGAGAGTGTGGT	2710
2711	**GTATTCCCCCAGCATGCGGGGGTATCGAC**		2739

(a)

CRISPR id : tmp_63_Crispr_1

- CRISPR start position : 5219 ---------- CRISPR end position : 6461 ---------- CRISPR length : 1242
- DR consensus : CCGATAACCCCCGCATGCGGGGGGAATAC
- DR length : 29 Number of spacers : 20

5219	**GTCGATACCCCCGCATGCTGGGGGAATAC**	ACCACACTCTCGCAGTGGTTCTCGCTCATGCC	5279
5280	**CCGATAACCCCCGCAAGCGGGGGGAATAC**	GCCCTGCACCTGTTTCGCAAAATCCGAACCGG	5340
5341	**CCAATAACCCCCGCATGCGGGGGGAGTAC**	GTGGCGACCACCAGCGTCTTAGTGCAGGGAA	5400
5401	**CCGATAACCCCCGTACGCGGGGGGAATAC**	TATTCAGCCAGTTCTCCTGGGCACCGGGTCGG	5461
5462	**CCGATAACCCCCGCGTGCGGGGGGAATAC**	GAACAGGTCCCAATTCTCCGTCAGCAGCCTCA	5522
5523	**CCGATAACCCCCGCATGCGGGGGGAATAC**	AGCAGGTGCGCGTCAGAGTGCGCAGGCTCATG	5583
5584	**GCGATAACCCCCGTAAGCGGGGGGGGAAT**	ACCATTTTTCAAACAGCCGGATTTTCGATTTGCT	5646
5647	**CCGATAACCCCCGCAGTCGGGGGGGAATA**	CATCACCACACTCATAAATTAACAAAACATTAA	5708
5709	**CCGATAACCCCCGCACACGGGGGGAATAC**	CTGCACTGCCTGTCACTATTCCTCGAACTGCT	5769
5770	**CCGATAACCCCCGCAGACGGGGGGAATAC**	CGACACGGTAGACAACAATATTTGCTGATTTA	5830
5831	**CCGATAACCCCCGCAAGCGGGGGAATACG**	AGAGCCATCGACGTTTTGCCAGTATAACCTG	5890
5891	**CCGATAACCCCCGCATGCGGGGGGAATAC**	GAAACGACCTGGCGCAGAAACTTCAAATTGTT	5951
5952	**CCGATAACCCCCGTAAGCGAGGGGAATAC**	CCTGACGACATCGGATCACATTTGCGTTACCG	6012
6013	**CCGATAACCCCCGCATGCGGGGGGAATAC**	TACTAACAATATTTAACGAACGCCAACACACA	6073
6074	**CCGATAACCCCCGCATGCGGGGGGAATAC**	CACCGCCGTTACTTTGCTTTACTCAATCTCGG	6134
6135	**CCGATAACCCCCGCACACGGGGGGAATAC**	AAAAGACGTTTTTATGAAGGTTGCCGTTTTCG	6195
6196	**CCGATAACCCCCGCATGCGGGGAGAATAC**	GCCGCAGTGGATTTCCACTGATGTT	6249
6250	**CCGATAACCCCCGCATGCGGGGGGAATAC**	ATTTACAAATGAAGATTTTTCCCCATTGGTAA	6310
6311	**CCAATAACCCCCGCACACGGGGGGAATAC**	TTCCAGCCAGCGGGTAACCGTGGACAACACGA	6371
6372	**CCGATAACCCCCGCACACGGGGGGAATAC**	TTCCAGCCAGCGGGTGACGGTTGAGAGCACCA	6432
6433	**CCGATAACCCCCGCACACGGGGGGAATAC**		6461

(b)

Supplementary Figure 4

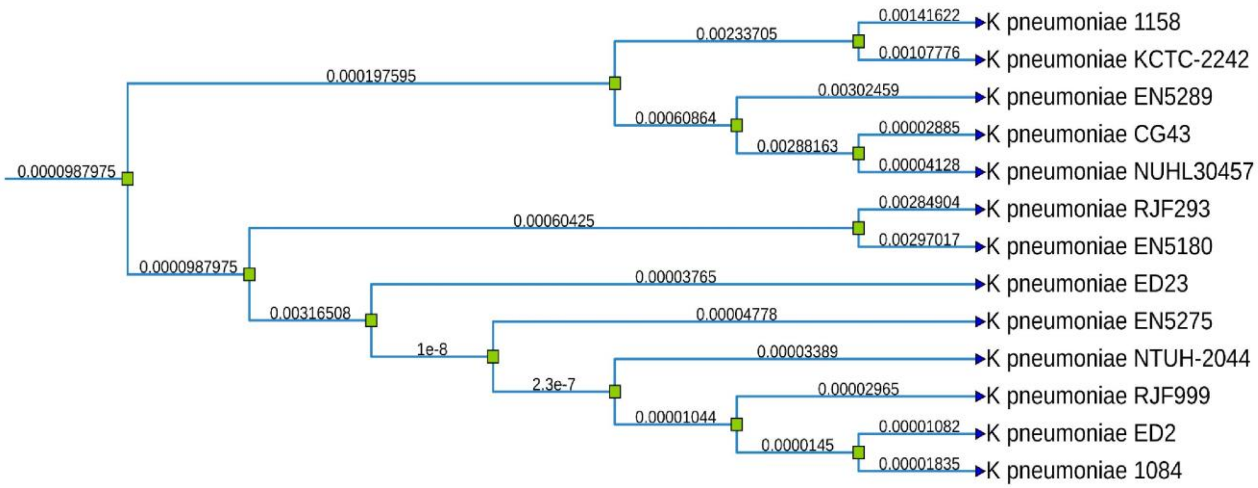

Strain ID	GenBank accession	Sequence type (ST)	Capsular serotype	Country of origin
1158	CP006722.1	65	K2	Taiwan
KCTC-2242	CP002910.1	375	K2	Korea
EN5289	JAELUW000000000	11	K2	India
CG43	CP006648.1	86	K2	Taiwan
NUHL30457	CP026586.1	86	K2	China
RJF293	CP014008.1	374	K2	china
EN5180	JAELUV000000000	15	K54	India
ED23	CP016814.1	23	K1	Taiwan
EN5275	VINI00000000	23	K1	India
NTUH-2044	NC_012731.1	23	K1	Taiwan
RJF999	CP014010.1	23	K1	China
ED2	CP016813.1	23	K1	Taiwan
1084	CP003785.1	23	K1	Taiwan

Supplementary Figure 5
